# Comparative Effectiveness of East Asian Traditional Medicine for Childhood Simple Obesity: A Systematic Review and Network Meta-Analysis

**DOI:** 10.3390/ijerph192012994

**Published:** 2022-10-11

**Authors:** Boram Lee, Chan-Young Kwon

**Affiliations:** 1 KM Science Research Division, Korea Institute of Oriental Medicine, Daejeon 34054, Korea; 2 Department of Oriental Neuropsychiatry, Dong-Eui University College of Korean Medicine, Busan 47227, Korea

**Keywords:** simple obesity, children, East Asian traditional medicine, network meta-analysis, systematic review

## Abstract

Childhood obesity leads to various comorbidities and usually persists into adulthood, increasing socioeconomic burden. In the absence of a clearly effective treatment, East Asian traditional medicine (EATM) therapies have been widely used. We aimed to analyze the comparative effectiveness and safety of EATM techniques for children with simple obesity through network meta-analysis (NMA). Twelve databases were searched for randomized controlled trials (RCTs) evaluating the effect of EATMs on childhood simple obesity. Individual EATMs were ranked based on the surface under the cumulative ranking curve. The risk of bias in the individual studies and publication bias in the NMA were evaluated. Thirty-three RCTs were included. Acupuncture, chuna, chuna plus acupressure, cupping plus acupressure, herbal medicine (HM), and HM plus acupuncture significantly reduced BMI compared with lifestyle management. Based on the treatment ranking, cupping plus acupressure was optimal for BMI reduction compared with a non-medical management, followed by chuna and HM. The quality of evidence for individual findings was usually moderate to low, and no serious adverse events of EATM were reported. Cupping plus acupressure might have a large beneficial effect, and chuna or HM probably have a moderate beneficial effect on reducing BMI in children with simple obesity.

## 1. Introduction

COVID-19 has had a profound impact not only on human health, but also on our lifestyle [1]. Dynamic lifestyle changes in individuals due to COVID-19 are associated with weight gain, reduced physical activity, and an increased intake of unhealthy foods [1]. In this context, the prevalence of obesity is gradually increasing in the era of COVID-19, which some scholars have termed “covibesity” [2]. The main victims of covibesity are children and adolescents, and their altered nutrition behavior, the marketing trends of an unhealthy food industry, insufficient physical activity, poor mental health, and household resilience are potentially involved [3,4,5]. Even before this pandemic, childhood obesity has been considered a serious public health crisis and has since been reported to be associated with poor health outcomes such as adult diabetes, hypertension, and sleep disturbances [6]. Simple obesity is prevalent in children, and according to a recent study in Massachusetts, the prevalence of obesity in children aged 6–11 years before the pandemic was 16.5% (2018–2019), but after the pandemic it increased to approximately 19% (2020) [7]. Eighty percent of childhood obesity is known to translate to adult obesity, and the annual direct cost of childhood obesity in the United States is estimated at approximately 14.3 billion USD [8]. In addition to these immediate costs, childhood obesity has a significant socioeconomic impact, given that it can lead to adult obesity in the future.

Although it is important to prevent children and adolescents being overweight and to prevent obesity, the preventive effect of behavioral prevention interventions for being overweight and obese in this population may be small, and there are limitations in the way that long-term benefits do not persist [9]. Likewise, behavioral interventions, including lifestyle management, have been mainly studied for treating overweight and obese children and adolescents, and there is low-to-moderate evidence that behavioral therapies can reduce body mass index (BMI) to a medium level [10]. On the other hand, clinical studies examining the efficacy and safety of pharmacotherapies for obesity in this population are limited [10], and a few drugs, including phentermine and orlistat, have been approved by the USA Food and Drug Administration for the treatment of adolescent obesity [11]. According to the recent clinical practice guidelines for the diagnosis and treatment of pediatric obesity, pharmacotherapies can reduce BMI (metformin, sibutramine, and orlistat) and waist circumference (WC) (sibutramine and orlistat), as well as increase high-density lipoprotein cholesterol (sibutramine) and systolic and diastolic blood pressure (sibutramine) [12]. However, as pharmacotherapies carry a risk of side effects such as gastrointestinal side effects, irritability, insomnia, and mood changes, these treatment strategies are considered an alternative treatment option for patients who have undergone and failed lifestyle-based therapies [11,12].

East Asian traditional medicine (EATM) is a medical system that includes the holistic use of treatment modalities such as acupuncture, moxibustion, herbal medicine (HM), massage, chuna, and acupressure, which are mainly used in China, Japan, Korea, and Taiwan [13,14], and may provide a promising alternative for treating obesity in children and adolescents. For example, a recent systematic review of eight randomized controlled clinical trials (RCTs) found that acupuncture had a significantly better effect on improving simple obesity than sham acupuncture [15]. There are also an increasing number of studies on the use of HM for the treatment of obesity [16]. Currently, various combinations of EATM therapies including HM, acupuncture, and moxibustion have been used in clinical settings for pediatric obesity. However, clinical studies directly comparing their effect sizes are rare. Therefore, a network meta-analysis (NMA) that can compare and rank the effectiveness and safety of various interventions through direct and indirect comparisons can help to establish an optimal treatment strategy [17]. However, to the best of our knowledge, objective evidence for the comparative effectiveness and safety of EATMs has not been systematically accumulated; therefore, there are not enough data for clinicians, patients, and policy makers to refer to for decision-making.

In this regard, the purpose of this study was to summarize and analyze the effects of EATM techniques compared with conventional treatment and to determine the comparative effectiveness and safety of EATM techniques for children with simple obesity through NMA.

## 2. Materials and Methods

### 2.1. Protocol Registration

The protocol of this study was registered with PROSPERO (CRD42022351514) prior to conducting the study, and we performed this study according to the protocol. This review complied with the PRISMA extension statement for the reporting of systematic reviews incorporating the NMA of health care interventions [18].

### 2.2. Eligibility Criteria

#### 2.2.1. Type of Study Design

Only parallel arm design RCTs were included. Cluster RCTs and crossover trials were excluded to avoid any potential bias. Although the specific method of randomization was not mentioned, all studies described as having an RCT design in the original text were included. There were no limitations regarding publication status, publication language, and publication year.

#### 2.2.2. Type of Population

We included RCTs involving pediatric patients with simple obesity who were younger than 19 years of age, without restrictions on their sex, race, and ethnicity. If a specific organic disease causing obesity was identified, the study was excluded.

#### 2.2.3. Type of Treatment Intervention

We set individual EATM therapies (including HM, acupuncture, acupressure, chuna, moxibustion, cupping therapy, qigong, Tai Chi, pharmacopuncture, bee venom acupuncture, and meditation) and a combination of these therapies as treatment interventions considering their applications in the clinical field and the definition of EATM in our previous study [13]. Among them, acupressure and chuna were mixed and used under various definitions in the included studies; thus, we defined acupressure as the static pressure of acupuncture points without needle penetration, and we defined chuna as pressure with movement.

#### 2.2.4. Type of Control Intervention

Placebo EATM therapies listed as the treatment interventions, conventional medical treatment, and a non-medical management (e.g., lifestyle management, such as regular exercise and nutritional supplementation), were considered to be the control interventions.

#### 2.2.5. Type of Outcome Measure

We included only studies reporting one or more of the following primary or secondary outcomes. The primary outcomes measured were post-treatment body weight-related anthropometric indicators including BMI, BMI percentile (of same age and sex), body weight, and body weight percentile (of same age and sex). The secondary outcome measures were (1) the post-treatment WC, hip circumference (HC), and waist–hip ratio (WHR), (2) post-treatment body fat mass, body fat ratio, and skeletal muscle mass, (3) post-treatment height and height percentile (of same age and sex), (4) total effective rate (TER) according to the improvement of obesity and related symptoms, and (5) quality of life.

For the units of analysis in the outcome assessment, we considered the first assessment after the end of the treatment. If a study was published in more than one journal, we included the more comprehensive and detailed report. Studies where only the abstract could be confirmed and were without data or the mean and standard deviation (SD) values of the outcomes of interest for our study were not specified in the full text, were excluded.

### 2.3. Information Sources and Search Strategy

The following four English, four Korean, three Chinese, and one Japanese electronic databases were searched comprehensively by one author (B Lee) on 1 July 2022: Medline, EMBASE, the Cochrane Central Register of Controlled Trials, the Allied and Complementary Medicine Database, the Oriental Medicine Advanced Searching Integrated System, the Korean Medical Database, the Korean Studies Information Service System, ScienceON, the China National Knowledge Infrastructure, Wanfang data, Chongqing VIP, and CiNii. To include as many studies as possible including gray literature, the reference lists of the relevant studies and the World Health Organization (WHO) International Clinical Trials Registry Platform were searched. The search strategy for the individual databases was established after discussions with experts in pediatrics and a systematic review, and detailed search strategies and the search results from each database are described in Supplementary S1.

### 2.4. Study Selection and Data Collection

Two authors (B.L. and C.Y.K.) independently conducted the study selection and data extraction process. In the case of a disagreement regarding the inclusion of a specific study or a disagreement during the data extraction, an agreement was reached through a discussion among the authors.

After removing duplicate records found in databases and other sources using EndNote 20 (Clarivate Analytics, Philadelphia, PA, USA), the eligible records were initially screened by reviewing the titles and abstracts. At this stage, full texts were retrieved for suitable records, and the appropriate studies were finally selected through a full-text review. The following information was extracted for the included studies using a pilot-tested Excel form: the basic study characteristics (the first author’s name, publication year, publication language, country where the study was conducted, study setting, and funding source), details about the populations (sample size, age, and pattern identification), treatment and control interventions, outcomes of interest, and adverse events. In cases where relevant information was unclear or vague even in the full-text review, the authors of the study were contacted via e-mail.

### 2.5. Risk of Bias within Individual Studies

The two authors (B.L. and C.Y.K.) independently assessed the risk of bias within individual studies using the Cochrane Collaboration’s risk of bias tool [19]. Any discrepancies were resolved through discussions between them. The tool assesses the following seven items including random sequence generation, allocation concealment, blinding of participants and personnel, blinding of outcome assessment, incomplete outcome data, selective reporting, and other biases. Each item was evaluated as a “low”, “unclear”, or “high” risk of bias. 

### 2.6. Data Analysis and Synthesis

#### 2.6.1. Qualitative Analysis

The main characteristics of the all included studies were qualitatively extracted and summarized.

#### 2.6.2. Conventional Pairwise Meta-Analysis

First, for studies with a clinically sufficiently homogeneous population, treatment and control intervention, and outcome measure, a conventional pairwise meta-analysis for direct comparisons was performed using Review Manager version 5.4 software (Cochrane, London, UK). Dichotomous data are presented as relative risks (RRs) with a 95% confidence interval (CI), and continuous data are reported as the weighted mean difference (MD) with a 95% CI. Statistical heterogeneity between studies was calculated based on the I^2^ value, and when the I^2^ value was 50 or higher, it was determined that there was substantial heterogeneity, and a random-effects model was used for pairwise meta-analysis. Otherwise, a fixed-effects model was used.

#### 2.6.3. NMA

Then, considering both direct and indirect effect estimates, an NMA was conducted based on the frequentist model using the ‘mvmeta’ package of Stata/MP software version 16 (StataCorp LLC., College Station, TX, USA). Considering the unavoidable clinical heterogeneity between studies, an NMA was performed based on a random-effects statistical model. Before performing NMA, clinical similarity and transitivity were evaluated, and consistency was tested through the node-splitting method (local approach) and design by the treatment interaction model (global approach). Transitivity and consistency can only be investigated empirically in networks with closed loops; therefore, we did not perform an NMA when the networks had no closed loops or contained few studies. For our outcomes of interest, a network map with the number of populations as the node size and the number of trials as the thickness of the lines was used to visually represent the whole intervention types and their comparisons included in each NMA.

For the purpose of our research, NMAs for various EATM therapies were conducted by grouping them into intervention types such as HM. In addition, for studies in which HM was used as a monotherapy, the comparative effects on the primary outcomes of individual HMs used were calculated as a subgroup analysis. When 10 or more studies were included in the meta-analysis, the risk of publication bias was visually confirmed through funnel plots and additionally tested through Egger’s test. We examined and ranked each intervention based on the surface under the cumulative ranking curve (SUCRA) to identify the best treatment. The higher the SUCRA value (closer to 100%), the higher the likelihood that a therapy is one of the top ranks; the closer the SUCRA value is to 0, the more likely that a therapy is one of the bottom ranks.

### 2.7. Quality of Evidence

The quality of evidence for direct (i.e., pairwise meta-analysis), indirect, and mixed (i.e., NMA) effect estimates derived for our primary and secondary outcomes was evaluated using the Grading of Recommendations, Assessment, Development, and Evaluations (GRADE) approach [20]. The risk of bias, indirectness, inconsistency, imprecision, and publication bias for the main findings were evaluated to rate the direct estimate. The lowest ratings of the two direct comparisons forming the most dominant first-order loop and intransitivity were considered to rate the indirect estimate. The higher rating of the direct or indirect estimates was determined as the quality of evidence rating for the NMA, and each rating is presented as “high”, “moderate”, “low”, or “very low”. In addition, for our primary outcomes, conclusions from the NMAs were drawn by considering the effect estimates, quality of evidence (according to the GRADE approach), and treatment rankings (i.e., SUCRA) based on the GRADE partially contextualized framework [21].

## 3. Results

### 3.1. Study Selection

**Figure 1:**
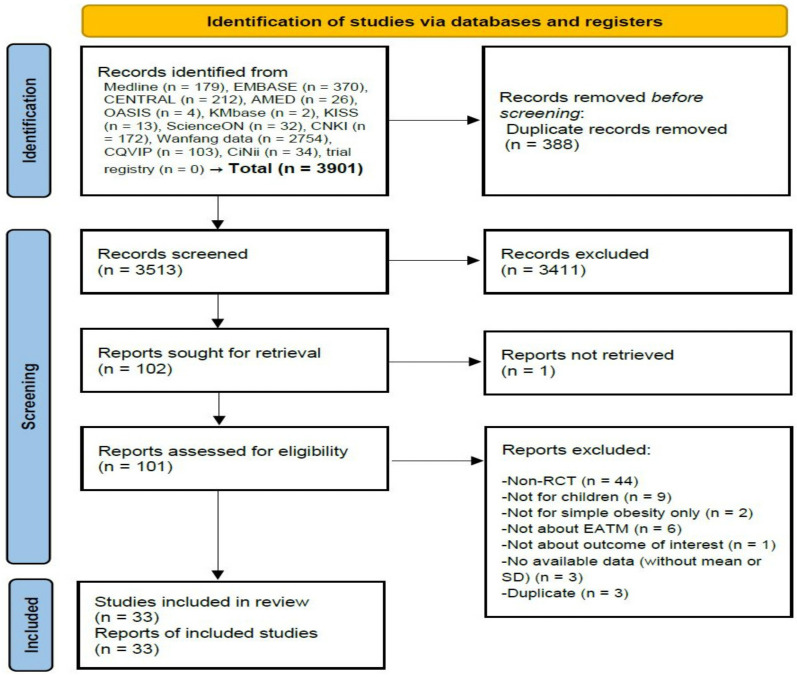
A PRISMA flow diagram of the literature screening and selection processes. AMED = Allied and Complementary Medicine Database, CENTRAL = Cochrane Central Register of Controlled Trials, CNKI = China National Knowledge Infrastructure, EATM = East Asian traditional medicine, KISS = Koreanstudies Information Service System, KMbase = Korean Medical Database, OASIS = Oriental Medicine Advanced Searching Integrated System, RCT = randomized controlled clinical trial, and SD = standard deviation.

A total of 3901 records were searched through the electronic database, and there were no identified records in the registry and other sources. After removing 388 duplicate records, 3513 records were screened for titles and abstracts, and 3411 records were excluded. A full-text review was performed on 101 of the remaining 102 records, except for one for which the full text was not retrieved. In this process, a total of 68 records were excluded for the following reasons: non-RCT design used (*n* = 44), pediatric patients not included (*n* = 9), simple obesity alone not evaluated (*n* = 2), EATM interventions not used (*n* = 6), outcomes of interest not reported (*n* = 1), no available data on effectiveness or efficacy (*n* = 3), and duplicates (*n* = 3) (Supplementary S2). Therefore, a total of 33 RCTs [22,23,24,25,26,27,28,29,30,31,32,33,34,35,36,37,38,39,40,41,42,43,44,45,46,47,48,49,50,51,52,53,54] were included in this review (Figure 1).

### 3.2. Study Characteristics

Of the 33 studies, 31 were conducted in China and two [23,24] were conducted in Korea. Three studies [22,23,24] were written in English, and the rest were published in Chinese. A total of 14 studies [22,23,24,33,38,42,44,46,47,48,49,50,51,53] reported funding sources, all of which received national or province academic research funding. As for the study setting, one study [22] targeted community-dwelling children, three studies [23,24,50] were conducted in schools, and the remaining studies were conducted in hospital or clinic settings. There were 17 studies [22,26,27,29,31,33,34,35,37,38,40,44,45,48,49,53,54] that reported participants’ pattern identification. Most of them were related to the spleen, stomach, and dampness, and pattern identification related to liver depression and Qi stagnation was also reported.

The interventions in the treatment groups include EATM monotherapy, such as acupuncture, acupressure, moxibustion, HM, chuna, and cupping as well as combined EATM treatments such as chuna plus acupressure, chuna plus acupuncture, cupping plus acupressure, HM plus acupuncture, and HM plus acupressure. As interventions in the control group, a non-medical management was used the most in 24 studies [22,25,26,27,28,29,30,32,33,34,35,38,39,40,41,42,43,46,47,48,49,50,52,53], placebo was used in two studies [23,24], and fenfluramine, a conventional medication, was used in one study [36]. The remaining six studies [31,37,44,45,51,54] compared the effects between EATMs. Two studies compared the effects of two different acupuncture [30] or chuna [38] methods with a non-medical management. We calculated the effects of the acupuncture or chuna methods used by synthesizing them. Six studies [23,25,32,41,47,51] reported that they had received approval from an institutional review board before conducting the study (Supplementary S3). 

### 3.3. Details of EATM Interventions Used in Individual Studies

A total of 15 studies [25,34,35,36,37,41,43,44,45,46,47,48,49,51,53] used HM as an intervention. Among them, Erchen tang was used the most commonly in a total of four studies [45,46,49,51], followed by Fangfeng Tongsheng pill (three studies [37,44,53]) and modified Wendan tang (two studies [25,47]). In one study [34], various HMs were used according to the participant’s pattern identification. With regard to the dosage form, a decoction was the most common in nine studies [25,41,43,45,46,47,49,51,53]. The administration period ranged from 20 days to 3 months, and 12 weeks or 3 months was the most common in nine studies [25,35,36,41,44,47,48,49,53]. In three studies [25,45,51], herbs were added to the basic prescription according to specific symptoms or pattern identifications. When the constituents of HMs were analyzed, a total of 46 kinds of herbs were used, and among them, *Pinelliae Tuber* was used the most commonly in 11 studies, followed by *Citri Unshius Pericarpium* (10 studies), *Glycyrrhizae Radix et Rhizoma* (8 studies), *Poria Sclerotium* (8 studies), and *Zingiberis Rhizoma Recens* (5 studies) (Supplementary S4).

As a result of analyzing the details of non-pharmacological EATMs, acupressure was used in nine studies, of which eight [22,23,24,26,32,49,50,54] involved mostly auricular acupressure, and one [31] involved body acupuncture points. With regard to the acupressure time, the auricular points were most commonly stimulated for five minutes per session, and the body acupuncture points were stimulated for two hours. Acupressure was mainly performed 3–5 times a day using materials such as *Vaccaria seeds*. Acupuncture was used in a total of six studies, electroacupuncture in four studies [30,45,46,51], and manual acupuncture in two studies [27,29]. Acupuncture was mainly performed once every 1–2 days for a total of 20–70 days. Chuna was used in 11 studies [26,28,29,31,32,33,37,38,42,52,54] and was mainly performed once a day for 4–12 weeks. Cupping therapy was used for a total of 4 weeks for 5–7 days a week in two studies [31,40], and one of them [31] used fire cupping. In two studies [39,50], moxibustion was performed for 2–3 min per acupuncture point, once a day, for 3 months. As a result of analyzing the basic acupuncture points used in studies using non-pharmacological EATMs, a total of 55 acupuncture points including 38 body acupuncture points and 17 auricular acupuncture points were used. Among them, CV12 and ST25 were the most frequently used in 15 studies, followed by ST36 (12 studies), CV4 (9 studies), CV6 (9 studies), and endocrine (8 studies) (Supplementary S5).

### 3.4. Risk of Bias within Individual Studies

**Figure 2:**
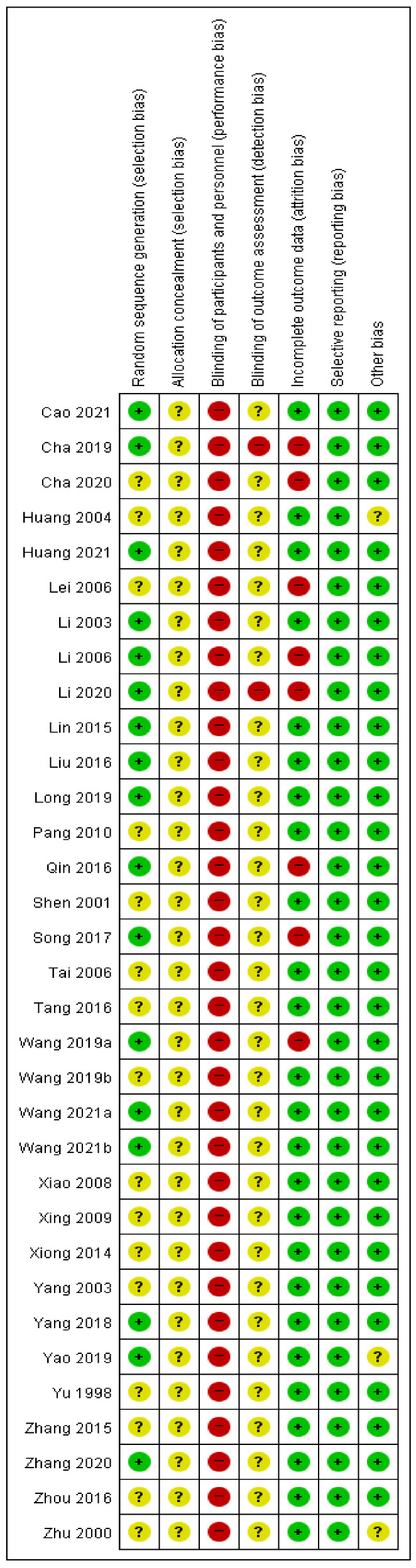
Risk of bias summary for all included studies. Low, unclear, and high risk of bias, respectively, are represented with the following symbols: “+”, “?”, and “−” [22,23,24,25,26,27,28,29,30,31,32,33,34,35,36,37,38,39,40,41,42,43,44,45,46,47,48,49,50,51,52,53,54].

Seventeen studies [22,23,25,28,29,30,31,32,33,35,37,40,42,43,47,49,51] were evaluated as being low risk in a random sequence generation domain because an appropriate random sequence generation method, such as a random number table, was used. No studies reported allocation concealment or blinding participants and personnel. In one study [23], only the study participants were blinded, and in the other study [29], blinding was not performed; therefore, these two studies were evaluated as high risk of detection bias. Eight studies were evaluated as having a high risk of attrition bias, of which seven studies [23,24,29,30,35,37,40] did not perform an intention-to-treat analysis, and one study [27] did not report the results of abdominal wall fat thickness and scapular fat thickness. All studies were evaluated as having a low risk of reporting bias, and three studies [26,49,54] that did not report on the statistical homogeneity of the baseline between the two groups, were evaluated as having an unclear risk of other bias (Figure 2).

### 3.5. Effects Derived from Pairwise Meta-Analysis and NMA

(1) BMI

**Figure 3:**
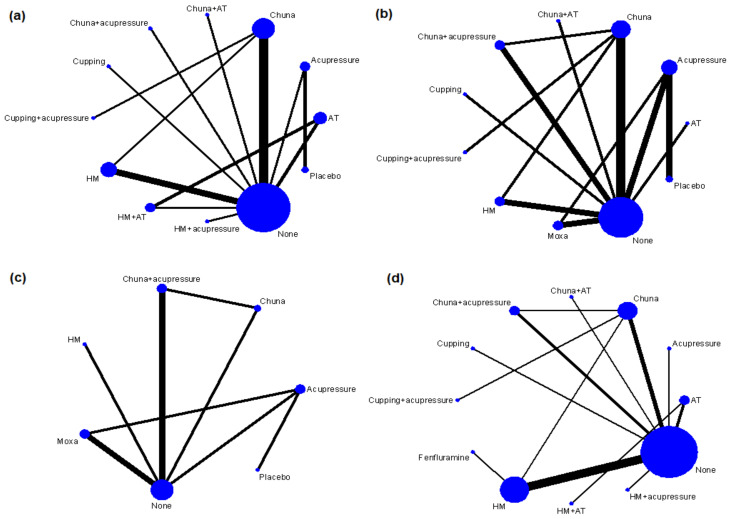
Network map of (**a**) body mass index, (**b**) body weight, (**c**) height, and (**d**) total effective rate. AT = acupuncture, HM = herbal medicine, Moxa = moxibustion, None = non-medical management.

A total of 23 studies were included in the NMA for BMI, and the following 12 interventions were included in the analysis: acupuncture, acupressure, chuna, chuna plus acupuncture, chuna plus acupressure, cupping, cupping plus acupressure, HM, HM plus acupuncture, HM plus acupressure, a non-medical management, and placebo (Figure 3a). In a pairwise meta-analysis, acupuncture, chuna, chuna plus acupuncture, chuna plus acupressure, HM, HM plus acupuncture, and HM plus acupressure significantly reduced BMI after treatment compared with a non-medical management, and there were no significant differences when using acupressure or cupping. However, in the NMA, only acupuncture, chuna, HM, HM plus acupuncture (significant difference), acupressure, and cupping (no significant difference) showed consistent results with the results of the pairwise meta-analysis. In addition, in the NMA, (a) cupping plus acupressure compared with a non-medical management, (b) chuna, cupping plus acupressure, and HM compared with placebo, (c) cupping plus acupressure compared with cupping or acupressure, and (d) HM or chuna compared with acupressure significantly decreased BMI after treatment (Table 1). According to the SUCRA, cupping plus acupressure was the optimal intervention for reducing BMI, followed by chuna, HM, chuna plus acupressure, and chuna plus acupuncture. However, a risk of publication bias was suggested based on Egger’s test (*p* = 0.007).

As a subgroup analysis, the effect difference according to the individual HM was investigated through NMA. Four HMs (modified Cangfu Daotan granule, Fangfeng Tongsheng pill, modified Wendan tang, and Jianfei oral liquid) from a total of five studies [35,37,44,47,53] were included in the analysis. As for the results, the Jianfei oral liquid significantly reduced BMI compared with other HMs (modified Cangfu Daotan granule: MD −7.83, 95% CI −10.07 to −5.59; Fangfeng Tongsheng pill: MD −2.30, 95% CI −2.97 to −1.63; modified Wendan tang: MD −7.49, 95% CI −9.42 to −5.56), and the Fangfeng Tongsheng pill also significantly reduced BMI compared with the modified Cangfu Daotan granule (MD −5.53, 95% CI −7.66 to −3.40) and the modified Wendan tang (MD −5.19, 95% CI −7.00 to −3.38). The SUCRA indicated that the Jianfei oral liquid was optimal for reducing BMI, followed by the Fangfeng Tongsheng pill, the modified Wendan tang, and the modified Cangfu Daotan granule.

(2) Body weight

A total of 18 studies were included in the NMA, and the following 11 interventions were included in the analysis: acupuncture, acupressure, chuna, chuna plus acupuncture, chuna plus acupressure, cupping, cupping plus acupressure, HM, moxibustion, a non-medical management, and placebo (Figure 3b). According to the pairwise meta-analysis, acupuncture, chuna, chuna plus acupuncture, chuna plus acupressure, and HM significantly reduced body weight after treatment compared with a non-medical management. However, in the NMA, only chuna, chuna plus acupressure, and HM showed significant results consistent with the results of the pairwise meta-analysis. In the NMA, cupping plus acupressure significantly reduced body weight compared with a non-medical management, and chuna, cupping plus acupressure, and HM significantly reduced body weight compared with placebo. In addition, HM, cupping plus acupressure, chuna plus acupressure, and chuna significantly reduced body weight after treatment compared with acupressure alone (Table 2). According to the SUCRA, cupping plus acupressure was the optimal treatment for reducing body weight, followed by HM, chuna, chuna plus acupressure, and chuna plus acupuncture. In the case of publication bias, the risk was evaluated as low by Egger’s test (*p* = 0.060). However, the network was not closed, and the number of studies was very limited; therefore, it was not possible to compare the effects between the individual HMs used.

(3) Height

A total of eight studies were included in the NMA, and the following seven interventions were included: acupressure, chuna, chuna plus acupressure, HM, moxibustion, a non-medical management, and placebo (Figure 3c). In both the pairwise meta-analysis and NMA, acupressure or moxibustion significantly increased the height after treatment compared with a non-medical management, and there was no significant difference in chuna, chuna plus acupressure, or HM compared with a non-medical management (Supplementary S6). The SUCRA indicated that moxibustion was optimal, followed by acupressure, placebo, and HM.

(4) Total effective rate (TER) according to an improvement in obesity and its related symptoms

A total of 23 studies were included in the NMA, and the following 12 interventions were analyzed: acupuncture, acupressure, chuna, chuna plus acupuncture, chuna plus acupressure, cupping, cupping plus acupressure, fenfluramine, HM, HM plus acupuncture, HM plus acupressure, and a non-medical management (Figure 3d). The TER was significantly higher for acupuncture, acupressure, chuna plus acupuncture, chuna plus acupressure, HM, HM plus acupuncture, and HM plus acupressure than for a non-medical management in the NMA. These results are consistent with the pairwise meta-analysis, except for the disappearance of the significance for chuna plus acupressure. In particular, the TER of HM plus acupuncture was significantly higher than that of other EATM interventions, except for HM plus acupressure, which had no significant difference (Supplementary S7). The SUCRA indicated that HM plus acupuncture was optimal, followed by acupuncture, HM plus acupressure, chuna plus acupuncture, chuna plus acupressure, and acupressure. There was a risk of publication bias in the asymmetry of the funnel plot and the results of Egger’s test (*p* = 0.001).

(5) Other outcomes of interest

Among the following secondary outcomes including WC, HC, WHR, body fat ratio, body fat mass, and skeletal muscle mass, only a pairwise meta-analysis was possible because the transitivity and consistency in the networks could not be examined. Acupressure significantly reduced WC and HC after treatment compared with a non-medical management (MD −1.10 cm, 95% CI −1.91 to −0.29; MD −1.20 cm, 95% CI −2.08 to −0.32). Compared with placebo (acupressure on five acupuncture points that have not been effective in obesity treatment), acupressure significantly decreased the WC (MD −3.95 cm, 95% CI −6.11 to −1.80), body fat ratio (MD −2.09%, 95% CI −3.88 to −0.31), and body fat mass (MD −1.82 kg, 95% CI −3.58 to −0.07); however, there was no significant difference in HC, WHR, and skeletal muscle mass. Compared with a non-medical management, chuna plus acupuncture (MD −2.00 cm, 95% CI −3.73 to −0.27), chuna (MD −3.22 cm, 95% CI −4.30 to −2.13), and HM (MD −5.98 cm, 95% CI −8.53 to −3.43) significantly reduced WC. In addition, chuna (MD −2.79%, 95% CI −5.57 to −0.01) or HM (MD −1.61%, 95% CI −1.91 to −1.31) significantly reduced the body fat ratio compared with a non-medical management. 

### 3.6. Adverse Events

A total of 12 studies [23,24,29,31,33,35,36,38,40,47,48,49] reported adverse events that occurred during the clinical trial period. Seven of them [24,29,31,33,35,40,49] reported that no adverse events occurred. One study [23] compared acupressure and placebo and reported two cases of minor itching of the skin where the tape was attached. In another study [36], adverse events did not occur in the HM group. However, in the fenfluramine group, four participants discontinued treatment due to obvious adverse events such as drowsiness affecting their learning, mental depression, and severe anorexia. Other participants also reported adverse events (e.g., dry mouth, dizziness, fatigue, and mild diarrhea). The other study [38] compared chuna and a non-medical management and reported that a transient increase in appetite occurred in one participant. One study [48] compared HM and a non-medical management and reported that no other adverse events were found except that the stool texture became thinner, the odor was obvious, and the stool frequency increased to twice a day. Another study [47] reported that there was one mild rash in the HM group and that no adverse events occurred in the non-medical management group (Supplementary S3).

### 3.7. Quality of Evidence

The quality of the direct (i.e., pairwise meta-analysis), indirect, and mixed (i.e., NMA) evidence evaluated according to the GRADE methodology for BMI, height, and TER after treatment was “low” to “moderate.” The reason for the downgrading was mainly due to the risk of bias in the included studies and imprecision due to wide CIs or small sample sizes. The quality of the direct (i.e., pairwise meta-analysis), indirect, and mixed (i.e., NMA) evidence for the post-treatment body weight was “very low” to “moderate.” In addition to the risk of bias and imprecision, the reason for the downgrading was the inconsistency of the effect estimate (Supplementary S8).

According to the GRADE partially contextualized framework, when considering all the interventions, cupping plus acupressure might have a large beneficial effect on reducing BMI and body weight. In addition, chuna or HM probably have a moderate beneficial effect, and chuna plus acupuncture might have a moderate beneficial effect on reducing BMI and body weight. Chuna plus acupressure might have a moderate beneficial effect on reducing BMI and probably has a moderate beneficial effect on reducing body weight. In addition, HM plus acupuncture or acupuncture probably has a moderate beneficial effect on reducing BMI. Furthermore, HM plus acupressure or acupuncture might have a small beneficial effect on reducing BMI or body weight, respectively (Table 3 and Table 4). 

## 4. Discussion

### 4.1. Summary of Evidence

We comprehensively searched a total of 12 electronic databases, and a total of 33 RCTs were included in this NMA. The following 11 EATMs were analyzed as interventions: acupressure, acupuncture, HM, chuna, moxibustion, cupping, chuna plus acupressure, chuna plus acupuncture, cupping plus acupressure, HM plus acupressure, and HM plus acupuncture. Among them, HM was used the most in 15 studies, chuna was used in 11 studies, and acupressure (9 studies) and acupuncture (6 studies) were also used frequently. We calculated the effect estimates between individual EATMs or compared them with conventional treatments through an NMA for the outcomes of interest and calculated the SUCRA-based treatment ranking. However, most of the evidence evaluated according to the GRADE approach was “moderate” to “low” in quality, and there was no high-quality evidence. For the primary outcomes, BMI and body weight, we drew conclusions in consideration of the effect estimates, quality of evidence, and treatment rankings based on the GRADE partially contextualized framework. As a result, cupping plus acupressure might have a large beneficial effect; chuna, HM, and HM plus acupuncture probably have a moderate beneficial effect; and chuna plus acupressure and chuna plus acupuncture might have a moderate beneficial effect on reducing BMI and/or body weight. Acupressure, cupping, and acupuncture showed a greater effect in reducing BMI and body weight when used in combination with other EATM treatments than when used as single treatments. Regarding the height after treatment, a secondary outcome, moxibustion was optimal, followed by acupressure according to the SUCRA. However, since this review did not target patients with a short stature, the results of the analysis on the height should be interpreted for reference only. Other secondary outcomes such as the WC, HC, and body fat ratio were not reported in most studies; therefore, only pairwise meta-analysis was possible. Some interventions such as acupressure, chuna, and HM have reported significant effects compared with a non-medical management. However, since most of the results were derived from one or two studies, caution is required in interpretation. Twelve studies reported safety after EATM therapies in children with simple obesity, and no serious adverse events were reported in the EATM group.

### 4.2. Clinical Interpretation

In improving the BMI of pediatric patients with obesity, although a combination of pharmacotherapy (e.g., orlistat) and behavioral interventions (e.g., lifestyle modifications) may be applicable, behavioral interventions are generally considered to be more effective [10]. In addition, in the case of pediatric patients with severe obesity, lifestyle modifications, pharmacotherapy, and bariatric surgery may be applied; however, lifestyle modifications have an insufficient efficacy, pharmacotherapy lacks research supporting its effectiveness, and bariatric surgery is accompanied by higher risks and a lower uptake [55]. Therefore, it is important to develop other effective options that can be used clinically in pediatric obese patients. The results of this review show that some EATM interventions might or probably have beneficial effects in reducing the BMI and/or body weight of obese pediatric patients. One of the important findings of this review is the importance of non-pharmacological treatments for this population. Specifically, the combination of acupressure and cupping was found to be the most effective in reducing both the BMI and body weight of obese pediatric patients. This is clinically relevant, given the preference for non-pharmacological strategies such as lifestyle modifications in childhood obesity [56].

Acupressure can be considered a noninvasive form of acupuncture, and its anti-obesity mechanisms may include neuroendocrine axis regulation. Specifically, this intervention has the potential to activate the satiety center and modulate appetite by regulating certain hormones, such as insulin and ghrelin [57]. The appetite-suppressing effect of acupressure has been consistently reported in previous studies [58,59,60]. Moreover, it has been reported that acupressure, especially auricular acupressure for 12 weeks, has a significant weight loss effect in overweight and obese people [61,62]. Cupping is also one of the most studied EATM interventions, and there is clinical evidence supporting its beneficial effects on herpes zoster, Bell’s palsy, acne, and cervical spondylosis [63]. Mechanisms explaining the effects of cupping therapy on obesity have been poorly studied, but the immunomodulatory effects of cupping therapy could potentially be related to its anti-obesity effects [64]. The clinical effects of cupping are considered to include modulating immunoglobulins, hemoglobin, and their various immunological effects, altering the local microenvironment, and activating the neuroendocrine immune system [64]. Recently, there is growing evidence that obesity is linked to inflammation and the immune system [65]. In particular, adipose tissue in obese patients is associated with more pro-inflammatory markers, and the patients may have chronic low-grade inflammation [65]. In this regard, cupping has the potential to help improve some indicators in pediatric obese patients through a modulation of the neuroendocrine immune system [66].

When applied as separate interventions, acupressure and cupping had an insignificant effects on pediatric obese patients; however, notably, the two interventions combined had the largest effect size. This is consistent with the results of a recent NMA showing that acupuncture and related therapies are more effective for obesity when used in combination than when used alone [67]. In addition, a clinical trial in China reported that the combination of two or more EATM interventions was more effective in the treatment of simple obesity [68]. However, the underlying mechanisms that explain the therapeutic effect of two or more interventions in pediatric obese patients and the optimal combination strategy still needs to be elucidated. Importantly, these non-pharmacological EATM interventions can be combined with existing behavioral interventions for obesity. For example, a recent retrospective case-controlled study reported a combination of aerobic exercise and acupuncture in obese patients [69]. However, effective combination methods, patient compliance, and their relative effects with existing behavioral interventions still need to be further studied.

### 4.3. Strengths and Limitations

This study was the first to comprehensively analyze the comparative effects of various EATM therapies that are actively used for the treatment of simple obesity in clinical settings, based on NMA. Its strengths include the evaluation of the related risks of bias, such as publication bias, using a thorough methodology and drawing conclusions based on the GRADE partially contextualized framework.

However, the following limitations are recognized in this review. First, the methodological quality of the studies included in this review was not optimal. Specifically, the included studies usually had an unclear risk of selection and detection bias and a high risk of performance bias. This affected the quality of evidence for the main findings evaluated by the GRADE. Second, we evaluated the publication bias for the analysis results based on the symmetry of the funnel plot and Egger’s test and found a high risk of bias in the BMI and TER. Moreover, we searched various electronic databases comprehensively without language restrictions; however, all the included studies were published in China and Korea. There were no studies published in other countries, and this might suggest a potential reporting bias. This potential publication bias may affect the validity of the EATM found in this review and the generalizability of the results. Third, among the included studies, none of the studies reported age and sex-specific z-scores or percentiles for the outcomes of interest including BMI, body weight, and height. Unlike adults, children continue to grow; thus, in the treatment of obese patients, the goal should be set in the context of age and sex rather than losing weight unconditionally [70,71]. Finally, we statistically tested consistency in the NMA analysis; however, clinical heterogeneity in individual EATM such as HM type or the acupuncture point used in each study was inevitable.

### 4.4. Suggestions for Further Studies

In future studies involving obese children, age and sex-specific z-scores or percentiles should be reported for BMI, body weight, and height. Future studies should also report quality of life after EATM treatment in obese children. Rather than being dangerous by itself, obesity causes various comorbidities and lowers the self-esteem of children and adolescents, thereby decreasing their quality of life [72]. Therefore, it is necessary to report changes in the quality of life of childhood obese patients after EATM treatment through future studies, and long-term studies such as a registry investigating BMI and comorbidities in adulthood should be conducted. Furthermore, an economic evaluation of EATM should be performed based on the reported effects on BMI, body weight, and quality of life after EATM treatment. This will be used as basic data to discover cost-effective and safe treatment options for childhood obesity, for which there is no clear treatment method, and to strengthen the coverage of EATM treatment.

## 5. Conclusions

This systematic review with an NMA found a better relative effectiveness of some EATM interventions, such as a combination of acupressure and cupping, in treating pediatric obesity. Specifically, cupping plus acupressure might have a large beneficial effect, and chuna or HM probably have a moderate beneficial effect on reducing BMI and body weight. Given the promise of EATM interventions and their potential for combination with existing behavioral interventions, further high-quality RCTs and long-term registry studies on the effects of EATM in children with simple obesity should be conducted.

## Figures and Tables

**Table 1:** League table for pairwise meta-analysis (right upper part) and NMA (left lower part) effect estimates: body mass index.

AT	-	-	-	-	-	-	-	**1.22 (0.79, 1.65)**	-	**−2.83 (−3.80, −1.86)**	-
−2.72 (−6.16, 0.72)	Acupressure	-	-	-	-	-	-	-	-	0.71 (−0.01, 1.43)	−0.83 (−1.72, 0.06)
1.72 (−0.64, 4.08)	**4.44 (1.22, 7.65)**	Chuna	-	-	-	2.38 (−0.30, 5.06)	**−1.41 (−2.29, −0.53)**	-	-	**−3.51 (−4.34, −2.67)**	-
0.69 (−2.80, 4.18)	3.41 (−0.71, 7.53)	−1.03 (−4.29, 2.24)	Chuna + AT	-	-	-	-	-	-	**−2.70 (−3.62, −1.78)**	-
1.06 (−2.82, 4.94)	3.78 (−0.67, 8.23)	−0.66 (−4.34, 3.02)	0.37 (−4.12, 4.86)	Chuna + acupressure	-	-	-	-	-	**−3.07 (−5.00, −1.14)**	-
−1.16 (−4.76, 2.44)	1.56 (−2.65, 5.77)	−2.88 (−6.26, 0.51)	−1.85 (−6.10, 2.40)	−2.22 (−6.80, 2.36)	Cupping	-	-	-	-	−0.85 (−2.13, 0.43)	-
4.10 (−0.44, 8.63)	**6.82 (1.79, 11.85)**	2.38 (−1.49, 6.25)	3.41 (−1.66, 8.47)	3.04 (−2.30, 8.38)	**5.26 (0.12, 10.40)**	Cupping + acupressure	-	-	-	-	-
1.15 (−1.19, 3.49)	**3.87 (0.66, 7.07)**	−0.57 (−2.33, 1.20)	0.46 (−2.80, 3.72)	0.09 (−3.58, 3.76)	2.31 (−1.07, 5.69)	−2.95 (−7.20, 1.31)	HM	-	-	**−3.50 (−5.70, −1.30)**	-
0.44 (−1.33, 2.21)	3.16 (−0.39, 6.71)	−1.28 (−3.80, 1.25)	−0.25 (−3.85, 3.35)	−0.62 (−4.60, 3.36)	1.60 (−2.11, 5.31)	−3.66 (−8.28, 0.96)	−0.71 (−3.22, 1.80)	HM + AT	-	**−0.88 (−1.61, −0.15)**	-
−0.79 (−4.31, 2.73)	1.93 (−2.22, 6.08)	−2.51 (−5.81, 0.80)	−1.48 (−5.67, 2.71)	−1.85 (−6.37, 2.67)	0.37 (−3.91, 4.65)	−4.89 (−9.97, 0.20)	−1.94 (−5.23, 1.35)	−1.23 (−4.87, 2.40)	HM + acupressure	**−1.22 (−2.27, −0.17)**	-
**−2.01 (−3.89, −0.13)**	0.71 (−2.17, 3.59)	**−3.73 (−5.15, −2.30)**	−2.70 (−5.64, 0.24)	−3.07 (−6.46, 0.32)	−0.85 (−3.92, 2.22)	**−6.11 (−10.23, −1.98)**	**−3.16 (−4.56, −1.76)**	**−2.45 (−4.53, −0.37)**	−1.22 (−4.20, 1.76)	None	-
−3.50 (−7.56, 0.57)	−0.78 (−2.95, 1.39)	**−5.21 (−9.09, −1.33)**	−4.19 (−8.84, 0.47)	−4.56 (−9.51, 0.40)	−2.34 (−7.07, 2.40)	**−7.59 (−13.07, −2.11)**	**−4.65 (−8.52, −0.78)**	−3.94 (−8.10, 0.23)	−2.71 (−7.39, 1.97)	−1.49 (−5.09, 2.12)	Placebo

Results are presented as the mean difference (95% confidence interval). The comparison must be read from left to right. A mean difference less than zero indicates that treatment on the left is favored in both pairwise and network meta-analyses. Bold value means a significant difference between the groups. AT = acupuncture, HM = herbal medicine, and None = non-medical management.

**Table 2:** League table for pairwise meta-analysis (right upper part) and NMA (left lower part) effect estimates: body weight.

AT	-	-	-	-	-	-	-	-	**−3.47 (−6.50, −0.44)**	-
−5.33 (−13.41, 2.75)	Acupressure	-	-	-	-	-	-	0.90 (−2.36, 4.16)	3.10 (−2.26, 8.46)	−1.48 (−5.17, 2.22)
2.22 (−5.82, 10.26)	**7.55 (1.32, 13.79)**	Chuna	-	0.80 (−6.27, 7.87)	-	**5.33 (0.49, 10.17)**	−2.68 (−11.80, 6.44)	-	**−5.35 (−9.15, −1.54)**	-
1.93 (−8.32, 12.18)	7.26 (-1.65, 16.17)	-0.29 (-9.16, 8.57)	Chuna + AT	-	-	-	-	-	**−5.40 (−10.22, −0.58)**	-
1.66 (−6.62, 9.94)	**6.99 (0.45, 13.53)**	−0.56 (−6.26, 5.13)	−0.27 (−9.35, 8.82)	Chuna + acupressure	-	-	-	-	**−4.78 (−8.05, −1.52)**	-
−1.93 (−12.59, 8.73)	3.40 (−5.97, 12.77)	−4.15 (−13.49, 5.18)	−3.86 (−15.16, 7.44)	−3.59 (−13.13, 5.95)	Cupping	-	-	-	−1.54 (−7.17, 4.09)	-
7.55 (−3.60, 18.71)	**12.88 (2.95, 22.82)**	5.33 (−2.40, 13.06)	5.62 (−6.14, 17.39)	5.89 (−3.71, 15.49)	9.48 (−2.64, 21.60)	Cupping + acupressure	-	-	-	-
2.39 (−6.11, 10.88)	**7.72 (0.90, 14.53)**	0.16 (−5.96, 6.28)	0.46 (−8.83, 9.74)	0.72 (−6.17, 7.62)	4.32 (−5.42, 14.05)	−5.17 (−15.03, 4.69)	HM	-	**−6.04 (−9.50, −2.58)**	-
−2.70 (−11.04, 5.64)	2.63 (−2.98, 8.24)	−4.92 (−11.47, 1.63)	−4.63 (−13.77, 4.51)	−4.36 (−11.21, 2.48)	−0.77 (−10.37, 8.82)	**−10.25 (−20.39, −0.12)**	−5.09 (−12.20, 2.02)	Moxa	−0.84 (−12.89, 11.21)	-
−3.47 (−10.22, 3.28)	1.86 (−2.59, 6.31)	**−5.69 (−10.06, −1.32)**	−5.40 (−13.12, 2.32)	**−5.13 (−9.93, −0.34)**	−1.54 (−9.79, 6.71)	**−11.02 (−19.90, −2.14)**	**−5.86 (−11.02, −0.69)**	−0.77 (−5.67, 4.13)	None	-
−6.77 (−16.69, 3.14)	−1.44 (−7.19, 4.30)	**−9.00 (−17.48, −0.52)**	−8.70 (−19.30, 1.90)	−8.44 (−17.14, 0.27)	−4.84 (−15.84, 6.15)	**−14.33 (−25.80, −2.85)**	**−9.16 (−18.07, −0.24)**	−4.07 (−12.10, 3.96)	−3.30 (−10.57, 3.96)	Placebo

Results are presented as the mean difference (95% confidence interval). The comparison must be read from left to right. A mean difference less than zero indicates that treatment on the left is favored in both pairwise and network meta-analyses. Bold value means a significant difference between the groups. AT = acupuncture, HM = herbal medicine, Moxa = moxibustion, and None = non-medical management.

**Table 3:** GRADE approach using a partially contextualized framework: body mass index.

Classification of Intervention	Intervention	Effect Estimates Compared with Non-Medical Management (MD (95% CI))	SUCRA (%)	Quality of Evidence
Large beneficial effect	Cupping + acupressure	−6.11 (−10.23, −1.98)	94.6	Low
Moderate beneficial effect	Chuna	−3.73 (−5.15, −2.30)	80.4	Moderate
	HM	−3.16 (−4.56, −1.76)	70.8	Moderate
	Chuna + acupressure	−3.07 (−6.46, 0.32)	67.3	Low
	Chuna + AT	−2.70 (−5.64, 0.24)	61.9	Low
	HM + AT	−2.45 (−4.53, −0.37)	58.8	Moderate
	AT	−2.01 (−3.89, −0.13)	50.2	Moderate
Small beneficial effect	HM + acupressure	−1.22 (−4.20, 1.76)	39.6	Low
Trivial or no effect	Cupping	−0.85 (−3.92, 2.22)	33.9	Low
	Acupressure	0.71 (−2.17, 3.59)	15.4	Low

AT = acupuncture, CI = confidence interval, HM = herbal medicine, MD = mean difference, and SUCRA = surface under the cumulative ranking probabilities.

**Table 4:** GRADE approach using a partially contextualized framework: body weight.

Classification of Intervention	Intervention	Effect Estimates Compared with Non-Medical Management (MD (95% CI))	SUCRA (%)	Quality of Evidence
Large beneficial effect	Cupping + acupressure	−11.02 (−19.90, −2.14)	93.1	Low
Moderate beneficial effect	HM	−5.86 (−11.02, −0.69)	71.6	Moderate
	Chuna	−5.69 (−10.06, −1.32)	71.1	Moderate
	Chuna + acupressure	−5.13 (−9.93, −0.34)	66.9	Moderate
	Chuna + AT	−5.40 (−13.12, 2.32)	66.1	Low
Small beneficial effect	AT	−3.47 (−10.22, 3.28)	53.7	Low
Trivial or no effect	Cupping	−1.54 (−9.79, 6.71)	40.7	Low
	Moxa	−0.77 (−5.67, 4.13)	34.4	Low
	Acupressure	1.86 (−2.59, 6.31)	15.2	Low

AT = acupuncture, CI = confidence interval, HM = herbal medicine, MD = mean difference, Moxa = moxibustion, and SUCRA = surface under the cumulative ranking probabilities.

## Data Availability

The datasets generated and analyzed for this study can be found in the manuscript and supplements.

## Supplementary Materials

The following supporting information can be downloaded at: https://www.mdpi.com/article/10.3390/ijerph192012994/s1, Supplementary S1: search strategies used in each database and the results; Supplementary S2: excluded studies after full-text review; Supplementary S3: general clinical characteristics of the included studies; Supplementary S4: details of HM used in the included studies; Supplementary S5: details of non-pharmacological EATMs used in the included studies; Supplementary S6: league table for pairwise meta-analysis (right upper part) and NMA (left lower part) effect estimates: height; Supplementary S7: league table for pairwise meta-analysis (right upper part) and NMA (left lower part) effect estimates: total effective rate; Supplementary S8: quality of evidence by the GRADE approach.
