# Supplementary material for: Comparative Effectiveness of East Asian Traditional Medicine for Childhood Simple Obesity: A Systematic Review and Network Meta-Analysis
Source: Int J Environ Res Public Health. 2022 Oct 11;19(20):12994. doi: 10.3390/ijerph192012994 (PMC9602315; doi:10.3390/ijerph192012994)
Supplement: Supplementary file 1 [file ijerph-19-12994-s001.zip › Supplement S1.pdf]

Supplement S1. Search strategies used in each database and the results

Medline via PubMed

	Searches	Results
#1	Obesity[MH] OR “Pediatric Obesity”[MH] OR Overweight[MH] OR Obesity[TIAB] OR Overweight[TIAB]	401788
#2	Child[MH] OR Pediatrics[MH] OR Infant[MH] OR Adolescent[MH] OR Minors[MH] OR child*[TIAB] OR pediatric*[TIAB] OR infant[TIAB] OR neonate[TIAB] OR newborn[TIAB] OR adolescent[TIAB] OR baby[TIAB]	4417050
#3	“Herbal Medicine”[MH] OR “Plants, Medicinal”[MH] OR “Drugs, Chinese Herbal”[MH] OR “Medicine, Chinese Traditional”[MH] OR “Medicine, Kampo”[MH] OR “Medicine, Korean Traditional”[MH] OR “traditional Korean medicine”[TIAB] OR “traditional Chinese medicine”[TIAB] OR “traditional oriental medicine”[TIAB] OR “Kampo medicine”[TIAB] OR herb*[TIAB] OR decoction*[TIAB] OR botanic*[TIAB] OR “Chinese patent medicine”[TIAB] OR Acupuncture[MH] OR “Acupuncture Therapy”[MH] OR Auriculotherapy[MH] OR “Acupuncture, Ear”[MH] OR Acupressure[MH] OR Electroacupuncture[MH] OR “Acupuncture Points”[MH] OR acupuncture[TIAB] OR acupressure[TIAB] OR acupoint*[TIAB] OR “trigger point”[TIAB] OR “dry needling”[TIAB] OR “ear acupuncture”[TIAB] OR electroacupuncture[TIAB] OR electro-acupuncture[TIAB] OR pharmacopuncture[TIAB] OR pharmaco-acupuncture[TIAB] OR “Bee Venoms”[MH] OR “bee venom*”[TIAB] OR “acupoint injection”[TIAB] OR Moxibustion[MH] OR moxibustion[TIAB] OR moxa[TIAB] OR “warm needling”[TIAB] OR “Tai Ji”[MH] OR “Tai Chi”[TIAB] OR “T’ai Chi”[TIAB] OR Taiji[TIAB] OR “Tai ji”[TIAB] OR taijiquan[TIAB] OR “Tai Chih”[TIAB] OR “Cupping Therapy”[MH] OR “cupping therapy”[TIAB] OR Qigong[MH] OR “qi gong”[TIAB] OR qigong[TIAB] OR “Breathing Exercises”[MH] OR Massage[MH] OR massage[TIAB] OR tuina[TIAB] OR chuna[TIAB] OR manipulat*[TIAB] OR “manual therapy”[TIAB] OR Chiropractic[TIAB] OR “Musculoskeletal Manipulations”[MH] OR “Manipulation, Spinal”[MH] OR “Manipulation, Chiropractic”[MH] OR “Meditation”[MH] OR “meditation”[TIAB]	533671
#4	“Randomized Controlled Trial”[PT] OR “Controlled Clinical Trial”[PT] OR randomized[TIAB] OR placebo[TIAB] OR “Clinical Trials as Topic”[Mesh: noexp] OR randomly[TIAB] OR trial[TI]	1549346
#5	animals[MH] NOT humans[MH]	5020991
#6	(#1 AND #2 AND #3 AND #4) NOT #5	**179**

EMBASE via Elsevier

	Searches	Results
#1	obesity/exp OR obesity:ab,ti OR overweight:ab,ti	732799
#2	child/exp OR pediatrics/exp OR infant/exp OR adolescent/exp OR ‘minor (person)’/exp OR child*:ab,ti OR pediatric*:ab,ti OR infant:ab,ti OR neonate:ab,ti OR newborn:ab,ti OR adolescent:ab,ti OR baby:ab,ti	4819292
#3	‘medicinal plant’/exp OR ‘medicinal plant’:ab,ti OR ‘plant medicinal product’/exp OR ‘plant medicinal product’:ab,ti OR ‘herbaceous agent’/exp OR ‘herbaceous agent’:ab,ti OR ‘chinese medicine’/exp OR ‘chinese medicine’:ab,ti OR ‘kampo medicine’/exp OR ‘kampo medicine’:ab,ti OR ‘kampo medicine (drug)’/exp OR ‘kampo medicine (drug)’:ab,ti OR ‘korean medicine’/exp OR ‘korean medicine’:ab,ti OR ‘herbal medicine’/exp OR ‘herbal medicine’:ab,ti OR ‘oriental medicine’/exp OR ‘oriental medicine’:ab,ti OR herb/exp OR herb:ab,ti OR decoction:ab,ti OR botanic:ab,ti OR ‘chinese patent medicine’:ab,ti OR acupuncture/exp OR acupuncture:ab,ti OR ‘acupuncture therapy’:ab,ti OR ‘auricular acupuncture’/exp OR ‘auricular acupuncture’:ab,ti OR ‘ear acupuncture’:ab,ti OR auriculotherapy:ab,ti OR acupressure/exp OR acupressure:ab,ti OR electroacupuncture/exp OR electroacupuncture:ab,ti OR ‘electro-acupuncture’:ab,ti OR ‘acupuncture point’/exp OR ‘acupuncture point’:ab,ti OR acupoint:ab,ti OR 'trigger point'/exp OR 'trigger point':ab,ti OR 'dry needling'/exp OR 'dry needling':ab,ti OR pharmacopuncture/exp OR pharmacopuncture:ab,ti OR ‘pharmaco-acupuncture’:ab,ti OR 'bee venom'/exp OR 'bee venom':ab,ti OR 'acupoint injection':ab,ti OR moxibustion/exp OR moxibustion:ab,ti OR moxa:ab,ti OR 'warm needling':ab,ti OR ‘Tai Chi’/exp OR ‘Tai Chi’:ab,ti OR Taiji:ab,ti OR ‘Tai ji’:ab,ti OR taijiquan:ab,ti OR ‘Tai Chih’:ab,ti OR 'cupping therapy'/exp OR 'cupping therapy':ab,ti OR qigong/exp OR qigong:ab,ti OR 'qi gong':ab,ti OR 'breating exercise'/exp OR 'breating exercise':ab,ti OR massage/exp OR massage:ab,ti OR tuina/exp OR tuina:ab,ti OR chuna:ab,ti OR 'manipulative medicine'/exp OR 'manipulative medicine':ab,ti OR 'manual therapy':ab,ti OR chiropractic/exp OR chiropractic:ab,ti OR 'musculoskeletal manipulation'/exp OR 'musculoskeletal manipulation':ab,ti OR 'spine manipulation'/exp OR 'spine manipulation':ab,ti OR 'chiropractic manipulation'/exp OR 'chiropractic manipulation':ab,ti OR meditation/exp OR meditation:ab,ti	1975302
#4	'crossover procedure':de OR 'double-blind procedure':de OR 'randomized controlled trial':de OR 'single-blind procedure':de OR (random* OR factorial* OR crossover* OR cross NEXT/1 over* OR placebo* OR doubl* NEAR/1 blind* OR singl* NEAR/1 blind* OR assign* OR allocat* OR volunteer*):de,ab,ti	2956803
#5	#1 AND #2 AND #3 AND #4	**370**

CENTRAL

	Searches	Results
#1	MeSH descriptor: [Obesity] explode all trees	15872

#2	MeSH descriptor: [Pediatric Obesity] explode all trees	1516
#3	MeSH descriptor: [Overweight] explode all trees	18989
#4	(obesity OR overweight):ti,ab,kw	46913
#5	#1 OR #2 OR #3 OR #4	47008
#6	MeSH descriptor: [Child] explode all trees	61534
#7	MeSH descriptor: [Pediatrics] explode all trees	727
#8	MeSH descriptor: [Infant] explode all trees	34961
#9	MeSH descriptor: [Adolescent] explode all trees	110346
#10	MeSH descriptor: [Minors] explode all trees	11
#11	(child* OR pediatric* OR infant OR neonate OR newborn OR adolescent OR baby):ti,ab,kw	301132
#12	#6 OR #7 OR #8 OR #9 OR #10 OR #11	301142
#13	MeSH descriptor: [Herbal Medicine] explode all trees	68
#14	MeSH descriptor: [Plants, Medicinal] explode all trees	952
#15	MeSH descriptor: [Drugs, Chinese Herbal] explode all trees	3800
#16	MeSH descriptor: [Medicine, Chinese Traditional] explode all trees	1274
#17	MeSH descriptor: [Medicine, Kampo] explode all trees	48
#18	MeSH descriptor: [Medicine, Korean Traditional] explode all trees	33
#19	MeSH descriptor: [Acupuncture] explode all trees	163
#20	MeSH descriptor: [Acupuncture Therapy] explode all trees	5285
#21	MeSH descriptor: [Auriculotherapy] explode all trees	254
#22	MeSH descriptor: [Acupuncture, Ear] explode all trees	216
#23	MeSH descriptor: [Acupressure] explode all trees	423
#24	MeSH descriptor: [Electroacupuncture] explode all trees	886
#25	MeSH descriptor: [Acupuncture Points] explode all trees	2248
#26	MeSH descriptor: [Bee Venoms] explode all trees	45
#27	MeSH descriptor: [Moxibustion] explode all trees	519
#28	MeSH descriptor: [Tai Ji] explode all trees	405
#29	MeSH descriptor: [Cupping Therapy] explode all trees	15
#30	MeSH descriptor: [Qigong] explode all trees	94
#31	MeSH descriptor: [Breathing Exercises] explode all trees	1001
#32	MeSH descriptor: [Massage] explode all trees	1286
#33	MeSH descriptor: [Musculoskeletal Manipulations] explode all trees	3342
#34	MeSH descriptor: [Manipulation, Spinal] explode all trees	429
#35	MeSH descriptor: [Manipulation, Chiropractic] explode all trees	138
#36	MeSH descriptor: [Meditation] explode all trees	743
#37	(“traditional Korean medicine” OR “traditional Chinese medicine” OR “traditional oriental medicine” OR “Kampo medicine” OR herb* OR decoction* OR botanic* OR “Chinese patent medicine” OR acupuncture OR acupressure OR acupoint* OR “trigger point” OR	64187

	“dry needling” OR “ear acupuncture” OR electroacupuncture OR pharmacopuncture OR pharmacoacupuncture OR “bee venom*” OR “acupoint injection” OR moxibustion OR moxa OR “warm needling” OR “Tai Chi” OR “T’ai Chi” OR Taiji OR “Tai ji” OR taijiquan OR “Tai Chih” OR “cupping therapy” OR “qi gong” OR qigong OR massage OR tuina OR chuna OR manipulat* OR “manual therapy” OR Chiropractic OR meditation):ti,ab,kw	
#38	#13 OR #14 OR #15 OR #16 OR #17 OR #18 OR #19 OR #20 OR #21 OR #22 OR #23 OR #24 OR #25 OR #26 OR #27 OR #28 OR #29 OR #30 OR #31 OR #32 OR #33 OR #34 OR #35 OR #36 OR #37	66049
#39	(“Randomized Controlled Trial” OR “Controlled Clinical Trial”):pt	640883
#40	(randomized OR placebo OR randomly OR trial):ti,ab,kw	1314752
#41	MeSH descriptor: [Clinical Trials as Topic] this term only	33290
#42	#39 OR #40 OR #41	1458619
#43	(#5 AND #12 AND #38 AND #42) in Trials	**212**

AMED via EBSCO

	Searches	Results
#1	Obesity[SU] OR “Pediatric Obesity”[SU] OR Overweight[SU] OR Obesity[TX] OR Overweight[TX]	3217
#2	Child[SU] OR Pediatrics[SU] OR Infant[SU] OR Adolescent[SU] OR Minors[SU] OR child*[TX] OR pediatric*[TX] OR infant[TX] OR neonate[TX] OR newborn[TX] OR adolescent[TX] OR baby[TX]	32793
#3	“Herbal Medicine”[SU] OR “Plants, Medicinal”[SU] OR “Drugs, Chinese Herbal”[SU] OR “Medicine, Chinese Traditional”[SU] OR “Medicine, Kampo”[SU] OR “Medicine, Korean Traditional”[SU] OR “traditional Korean medicine”[TX] OR “traditional Chinese medicine”[TX] OR “traditional oriental medicine”[TX] OR “Kampo medicine”[TX] OR herb*[TX] OR decoction*[TX] OR botanic*[TX] OR “Chinese patent medicine”[TX] OR Acupuncture[SU] OR “Acupuncture Therapy”[SU] OR Auriculotherapy[SU] OR “Acupuncture, Ear”[SU] OR Acupressure[SU] OR Electroacupuncture[SU] OR “Acupuncture Points”[SU] OR acupuncture[TX] OR acupressure[TX] OR acupoint*[TX] OR “trigger point”[TX] OR “dry needling”[TX] OR “ear acupuncture”[TX] OR electroacupuncture[TX] OR electro-acupuncture[TX] OR pharmacopuncture[TX] OR pharmaco-acupuncture[TX] OR “Bee Venoms”[SU] OR “bee venom*”[TX] OR “acupoint injection”[TX] OR Moxibustion[SU] OR moxibustion[TX] OR moxa[TX] OR “warm needling”[TX] OR “Tai Ji”[SU] OR “Tai Chi”[TX] OR “T’ai Chi”[TX] OR Taiji[TX] OR “Tai ji”[TX] OR taijiquan[TX] OR “Tai Chih”[TX] OR “Cupping Therapy”[SU] OR “cupping therapy”[TX] OR Qigong[SU] OR “qi gong”[TX] OR qigong[TX] OR “Breathing	66343

	Exercises"[SU] OR Massage[SU] OR massage[TX] OR tuina[TX] OR chuna[TX] OR manipulat*[TX] OR "manual therapy"[TX] OR Chiropractic[TX] OR "Musculoskeletal Manipulations"[SU] OR "Manipulation, Spinal"[SU] OR "Manipulation, Chiropractic"[SU] OR Meditation[SU] OR meditation[TX]	
#4	(#1 AND #2 AND #3)	**26**

OASIS

	Searches	Results
#1	(비만|과체중) (소아|아동) (한약|약초|본초|침|혈위|지압|봉독|뜸|태극권|부항|기공|마사지|추나|안마|명상)	**4**

KISS

	Searches	Results
#1	제목=(비만|과체중) AND 초록=(소아|아동) AND 초록=(한약|약초|본초|침|혈위|지압|봉독|뜸|태극권|부항|기공|마사지|추나|안마|명상)	**13**

KMbase

	Searches	Results
#1	([TITLE=비만] OR [TITLE=과체중])	3473
#2	([ABSTRACT=소아] OR [ABSTRACT=아동])	8924
#3	([ABSTRACT=한약] OR [ABSTRACT=약초] OR [ABSTRACT=본초] OR [ABSTRACT=침] OR [ABSTRACT=혈위] OR [ABSTRACT=지압] OR [ABSTRACT=봉독] OR [ABSTRACT=뜸] OR [ABSTRACT=태극권] OR [ABSTRACT=부항] OR [ABSTRACT=기공] OR [ABSTRACT=마사지] OR [ABSTRACT=추나] OR [ABSTRACT=안마] OR [ABSTRACT=명상])	11426
#4	#1 AND #2 AND #3	**2**

ScienceON

	Searches	Results
#1	(비만|과체중) (소아|아동) (한약|약초|본초|침|혈위|지압|봉독|뜸|태극권|부항|기공|마사지|추나|안마|명상)	**32**

CNKI

	Searches	Results
#1	(SU='**肥胖**'+'**超重**') AND (SU='小儿'+'儿童'+'儿'+'小孩'+'孩子') AND (SU='中药'+'中医药'+'本草'+'汤'+'丸'+'散'+'颗粒'+'胶囊'+'自拟'+’针’+’刺法‘+'指压'+'按压'+'耳压'+’穴位’+’蜂毒‘+’蜂疗‘+’穴位注射‘+’按摩'+'推拿'+'牵引'+'手法'+'太极拳'+'气功'+'灸'+'火罐'+'拔罐'+'冥想')	**172**

Wanfang data

	Searches	Results
#1	(主题:**肥胖** OR 主题:**超重**) AND (主题:小儿 OR 主题:儿童 OR 主题:儿 OR 主题:小孩 OR 主题:孩子) AND (主题:中药 OR 主题:中医药 OR 主题:本草 OR 主题:汤 OR 主题:丸 OR 主题:散 OR 主题:颗粒 OR 主题:胶囊 OR 主题:自拟 OR 主题:针 OR 主题:刺法 OR 主题:指压 OR 主题:按压 OR 主题:耳压 OR 主题:穴位 OR 主题:蜂毒 OR 主题:蜂疗 OR 主题:穴位注射 OR 主题:按摩 OR 主题:推拿 OR 主题:牵引 OR 主题:手法 OR 主题:太极拳 OR 主题:气功 OR 主题:灸 OR 主题:火罐 OR 主题:拔罐 OR 主题:冥想)	**2754**

CQVIP

	Searches	Results
#1	(M=(**肥胖 OR 超重)** AND M=(小儿 OR 儿童 OR 儿 OR 小孩 OR 孩子) AND M=(中药 OR 中医药 OR 本草 OR 汤 OR 丸 OR 散 OR 颗粒 OR 胶囊 OR 自拟 OR 针 OR 刺法 OR 指压 OR 按压 OR 耳压 OR 穴位 OR 蜂毒 OR 蜂疗 OR 穴位注射 OR 按摩 OR 推拿 OR 牵引 OR 手法 OR 太极拳 OR 气功 OR 灸 OR 火罐 OR 拔罐 OR 冥想))	**103**

CiNii

	Searches	Results
#1	(**肥胖 OR 超重**) AND (小児 OR 幼子 OR 児子 OR 子 OR 児 OR 児童 OR 幼児 OR 乳児 OR 子供 OR 思春期の OR 青春の) AND (ハーブ OR 漢方薬 OR 中药 OR 本草 OR 鍼 OR 刺法 OR 指圧 OR しあつ OR 按圧 OR 耳圧 OR 穴位 OR はちどく OR 蜂毒 OR 蜂疗 OR 穴位注射 OR マッサージ OR カイロプラクティック OR 按摩 OR 推拿 OR 牽引 OR 手法 OR 太極拳 OR 気功 OR 灸 OR 吸角 OR 拔罐 OR	**34**

	カッピング OR 冥想 OR 瞑想 OR めいそう)	
