# Supplementary material for: Comparative Effectiveness of East Asian Traditional Medicine for Childhood Simple Obesity: A Systematic Review and Network Meta-Analysis
Source: Int J Environ Res Public Health. 2022 Oct 11;19(20):12994. doi: 10.3390/ijerph192012994 (PMC9602315; doi:10.3390/ijerph192012994)
Supplement: Supplementary file 1 [file ijerph-19-12994-s001.zip › Supplement S2.pdf]

**Supplement S2. Excluded studies after full-text review**

**(1) Non-RCT (n=44)**

1. 康翠英 刘A潘A廖A姚A曹A. 中医推拿、耳穴按压合五行音乐治疗儿童单纯性肥胖61例总结. 湖南中医杂志. 2020;36(4).
2. 孔莎莎. 利湿活血法配合针刺治疗小儿单纯性肥胖症. 医学新知杂志. 2019;29(1):98-9.
3. 邱江东 邱A邱A. 自拟小儿减肥散治疗少年肥胖症160例. 辽宁中医杂志. 2000;27(1).
4. 김미숙, 이지원, 윤현민. 이침요법(耳鍼療法)이 아동의 비만도(肥滿度)에 미치는 효과(效果). 大韓鍼灸學會誌= The journal of Korean Acupuncture & Moxibustion Society. 2005;22(5):99-109.
5. 唐月荣, 段海洋. 缩食降脂颗粒(丸)治疗肥胖症的临床研究. 中国社区医师（医学专业）. 2010;12(17):124.
6. 董华, 林晏, 张祖仁, 章固. 气功治疗学生单纯性肥胖的观察. 气功杂志. 1999(08):43-4.
7. 廖燕霞, 刘芸, 邹秀齐. 梅州地区学龄前儿童肥胖群体的综合干预方法及效果分析. 中国医学工程. 2016;24(3):126-7.
8. 李芳. 清解化湿法配合耳穴贴压治疗儿童单纯性肥胖32例. 江西中医药. 2008(11):68.
9. 李海天, 张曼, 吕忠礼, 李时莲, 彭芸, 韩忠龙, et al. 针刺加电针治疗儿童单纯性肥胖症临床观察. 河北中医. 2011;33(9):1370-1.
10. 林连美 刘A. 中医药治疗小儿单纯性肥胖症的研究进展. 湖北中医杂志. 2013;35(5).
11. 莫娟, 欧阳俊. 儿童单纯性肥胖症的现状及中医治疗. 第四届国际中医糖尿病大会论文汇编; 中国北京2009.
12. 梅依君, 刘瑜, 涂林修, 王海燕, 顾世明, 陆荣耀. 六味地黄丸对单纯性肥胖儿童血内皮素的影响. 现代实用医学. 2014;26(06):720+9.
13. 宋亚飞. 推拿结合针刺治疗小儿单纯性肥胖26例观察. 内蒙古中医药. 2012;31(17):84-5.
14. 王迎春, 徐英杰. 指针结合耳穴及脉冲电刺激治疗小儿单纯性肥胖的临床观察. 特别健康：下. 2014(5):97-8.
15. 윤현민, 김미숙, 이지원. 耳鍼療法이 아동의 肥滿度에 미치는 效果. Journal of Acupuncture Research. 2005;22(5):99-109.
16. 殷云霞. 中医治疗小儿单纯性肥胖的护理体会. 现代养生（下半月版）. 2017(7):177.
17. 庄仁杰, 胡世东, 彭来恩, 兰天和. 针刺治疗儿童单纯性肥胖46例的临床观察. 实用中西医结合临床. 2008;8(3):66,74.
18. 翟巧玲. 基于心理疗法的综合干预措施与中医针灸治疗儿童单纯性肥胖疗效对比研究. 中华实用儿科临床杂志. 2014;29(13):1022-4.
19. 朱国礼. 全身调理结合局部消脂推拿法治疗小儿单纯性肥胖的临床分析. 光明中医. 2017;32(10):1475-7.
20. 曾裕宏, 韦怀籍, 祝秀文, 罗月中. 中医药治疗儿童单纯性肥胖症的研究进展. 中华现代儿科学杂志. 2005;002(4).
21. 曾艳, 胡国斌, 焦铁健, 潘永娴. 综合疗法治疗儿童单纯性肥胖症31例. 中国实用美容整形外科杂志. 2005;16(3):154-5.
22. 焦东海 沈A王A高A. 降脂减肥片治疗儿童单纯性肥胖症72例. 上海中医药杂志. 1997(7).

23. 沈小岚. 冬荷泻黄颗粒治疗儿童单纯性肥胖病的临床观察 [硕士]: 山东中医药大学; 2013.
24. 叶薇. 中药加味温胆汤治疗小儿单纯性肥胖效果分析. 中外医疗. 2020;39(04):152-4.
25. 胡文举. 穴位埋线结合耳穴贴压治疗单纯性肥胖53例. 中国民间疗法. 2011;19(12):24-5.
26. 冯晓纯 钱A王A钱A. 中医药治疗小儿单纯性肥胖症. 吉林中医药. 2017;37(9).
27. 刘怡湘. 耳压治疗儿童肥胖症93例临床分析. 中国针灸. 1990;010(3):14-5.
28. 刘长信, 王永学. 腹部针刺治疗单纯性肥胖100例临床观察. 北京中医药大学学报（中医临床版）. 2007;14(2):22-3.
29. 刘鹏 张A. 扶脾抑胃推拿法治疗儿童单纯性肥胖36例临床观察. 吉林医学信息. 2012;0(Z1).
30. 孙德仁, 陈博睿. 儿童肥胖症机理与少儿推拿综合调理. 实用中医内科杂志. 2020;34(01):96-9.
31. 孙升云, 杨钦河, 姚英民, 陈孝银. 利湿活血法治疗小儿单纯性肥胖症128例临床观察. 新中医. 2002;34(1):56-.
32. 孙雄. 温胆汤治疗小儿单纯性肥胖症初探. 第24届全国中医儿科学术研讨会、中医药高等教育儿科教学研讨会、儿科名中医讲习班论文汇编; 中国福建厦门2007.
33. 张跃红 琚A. 中医推拿对单纯性肥胖儿童血脂及性激素影响的临床观察. 中国民间疗法. 2015;23(5).
34. 罗璨璟, 孙远岭. 中药治疗单纯性肥胖症研究进展. 现代中西医结合杂志. 2010;19(31):3482-4.
35. 谢远军, 杨丹红, 詹红生, 解光尧. 推拿治疗106例单纯性肥胖症的临床研究. 按摩与导引. 2002;18(4):5.
36. 赵永利. 儿童单纯性肥胖症的基础与临床研究 [博士]: 大连医科大学; 2017.
37. 钱美加, 王丽丽, 卢琦, 冯晓纯. 中医药治疗小儿单纯性肥胖症研究进展. 中华中医药学会第34次全国中医儿科学术大会暨2017年广东省中西医结合儿科学术交流会论文集; 广州2017. p. 958-63.
38. 黄燕春. 中医推拿防治儿童肥胖症. 第五届国学国医岳麓论坛论文集; 长沙2011. p. 483-5.
39. Kapur R. Role of acupuncture in the treatment of obesity. International Journal of Clinical Acupuncture. 1999;10(1):9-14.
40. 高雅文. 温阳利水法治疗儿童肥胖症32例. 中国中医药信息杂志. 2001;8(12).
41. 段云雁. 瘦体合剂治疗儿童单纯性肥胖症的临床疗效观察及动物实验研究 [硕士]: 湖北中医学院; 2007.
42. 段云雁, 黄静宁. 瘦体合剂治疗儿童期单纯肥胖症的疗效观察. 湖北中医杂志. 2007(10):38.
43. 万梦婷, 徐达津, 龚婕, 徐海花, 胡苏平. 药罐结合干预疗法对33例单纯性肥胖症儿童体脂数、腹部脂肪厚度、血脂代谢影响的临床观察. 亚太传统医药. 2022;18(06):124-8.
44. 魏清琳, 达其伟, 潘虹, 寇萍萍, 金钰红, 王北平, et al. 经络点穴配合耳穴贴压治疗儿童单纯性肥胖症30例疗效观察. 甘肃中医学院学报. 2013;30(4):48-50.

**(2) not for children (n=9)**

1. 阮慧红. 穴位埋线治疗单纯性肥胖症的临床研究 [硕士]: 广州中医药大学; 2008.
2. 王静. 电针结合穴位埋线对脾虚湿阻型单纯性肥胖症的临床观察 [硕士]: 南京中医药大学; 2016.

3. 张红, 杨爱萍, 尹改珍. 穴位贴磁疗法对单纯性肥胖患者血脂、血糖影响的临床研究. 新疆中医药. 2005;23(6):26-7.
4. 罗珊娜. 刮痧结合针刺治疗单纯性肥胖临床观察 [博士]: 广州中医药大学; 2017.
5. 赵莉娟, 李晶, 刘小谓. 化痰减肥汤治疗单纯性肥胖病的临床研究. 中国医药学报. 2002;17(11):702-3.
6. 马丽君, 王晓平, 马腾. 经络穴位埋线及运动介入治疗单纯性肥胖的研究. 承德医学院学报. 2011;28(3):286-7.
7. Huang LC, Pan WY. [Comparation of effect and cost-benefit analysis between acupoint catgut-embedding and electroacupuncture on simple obesity]. Zhongguo Zhen Jiu. 2011;31(10):883-6.
8. Wang YL, Cao X, Liu ZC, Xu B. Observation on the therapeutic effect of electroacupuncture on simple obesity of gastrointestinal heat pattern/syndrome. World Journal of Acupuncture - Moxibustion. 2013;23(2):1-5.
9. Wei QL, Liu ZC. Treatment of simple obesity with auricular acupuncture, body acupuncture and combination of auricular and body acupuncture. Chinese Journal of Clinical Rehabilitation. 2004;8(21):4357-9.

**(3) not for simple obesity only (n=2)**

1. 沈小芬. 益气养阴疏肝方对单纯肥胖儿童糖耐量低减32例干预治疗的临床观察. 浙江中医药大学学报. 2007;31(3):334-5.
2. Yeh YC, Chen CC, Cheng CY, Chang HN, Huang TH. The Beneficial Effects of Moxibustion on Overweight Adolescent Girls. Evid Based Complement Alternat Med. 2021;2021:1943181.

**(4) not about EATM (n=6)**

1. 金亚军, 林建荣. 儿童单纯性肥胖症心理干预综合治疗的效果评价. 中国医药导报. 2008;5(34):163-4.
2. 路安洪 张A岳A刘A李A. 综合干预儿童单纯性肥胖群体:2年效果观察. 中国临床康复. 2004;8(24).
3. 刘丹 乐A王A夏A. 荷泽口服液治疗单纯性肥胖症患儿的临床观察. 中国中西医结合杂志. 2002;22(5).
4. 杨雪. 心理干预综合治疗对儿童单纯性肥胖症的短期效果评价. 健康必读（中旬刊）. 2013;12(11):353.
5. Tsang TW, Kohn M, Chin MC, Singh MF. A randomized controlled trial of Kung Fu training for metabolic health in overweight/obese adolescents: The "martial fitness" study. Journal of Pediatric Endocrinology and Metabolism. 2009;22(7):595-607.
6. Tsang TW, Kohn M, Chow CM, Singh MF. A randomised placebo-exercise controlled trial of Kung Fu training for improvements in body composition in overweight/obese adolescents: the "Martial Fitness" study. J Sports Sci Med. 2009;8(1):97-106.

**(5) not about outcome of interest (n=1)**

1. 张凤敏 谢A甘A. 耳针治疗儿童单纯性肥胖症35例疗效观察. 现代康复. 1999;3(2).

**(6) no available data (without mean or SD) (n=3)**

1. 徐布军 陈A钱A沈A. 337例儿童单纯性肥胖症的临床观察. 上海中医药杂志. 1992(12).
2. Hsieh CH. The effects of auricular acupressure on weight loss and serum lipid levels in overweight adolescents. Am J Chin Med. 2010;38(4):675-82.
3. Min J, Park H. Effects of Auricular Acupressure on Children with Obesity: randomized, double-blind, sham control. 한국간호과학회 학술대회. 2019.

**(7) duplicate (n=3)**

1. Le Q, Wang DX, Xia XH. [Clinical observation on effect of heze oral liquid in treating children simple obesity]. Zhongguo Zhong Xi Yi Jie He Za Zhi. 2002;22(5):384-5.
2. Yang C, Xin-yi W, Qing-yi Z, Gui-zhi M, Yin S, Jing Z. Efficacy observation of auricular point sticking in combination with a healthy diet for simple obesity in children. Journal of Acupuncture and Tuina Science. 2021;19(6):475-80.
3. Yu C, Zhao S, Zhao X. [Treatment of simple obesity in children with photo-acupuncture]. Zhongguo Zhong Xi Yi Jie He Za Zhi. 1998;18(6):348-50.
