# Supplementary material for: Comparative Effectiveness of East Asian Traditional Medicine for Childhood Simple Obesity: A Systematic Review and Network Meta-Analysis
Source: Int J Environ Res Public Health. 2022 Oct 11;19(20):12994. doi: 10.3390/ijerph192012994 (PMC9602315; doi:10.3390/ijerph192012994)
Supplement: Supplementary file 1 [file ijerph-19-12994-s001.zip › Supplement S3.pdf]

Supplement S3. General clinical characteristics of the included studies

Study ID	Sample size (enrolled →analyzed)	Age (mean ± SD) (yr)	Pattern identification	(A) Treatment intervention	(B) Control intervention	Duration of treatment / F/U	Outcome of interest	Adverse events
Cao 2021	190(95:95)	(A) 8.1 ± 0.3<br>(B) 7.3 ± 0.3	dampness stagnation due to Spleen deficiency, Stomach heat and dampness stagnation, and deficiency of Spleen and Kidney	Acupressure	Non-medical management	3 mon / none	1. BMI<br>2. Body weight<br>3. WC<br>4. HC<br>5. TER	NR
Cha 2019	68(34:34)<br>→58(32:26)	(A) 16.09 ± 6.0<br>(B) 15.96 ± 5.96	NR	Acupressure	Placebo<br>(five acupuncture points that have not been effective in obesity treatment were selected)	8 wk / none	1. BMI<br>2. Body weight<br>3. WC<br>4. WHR<br>5. Body fat ratio<br>6. Body fat mass (kg)	Minor itching on the skin when the tape was attached during auricular acupressure (2 cases, not specified which of the two groups)
Cha 2020	70(35:35)<br>→65(31:34)	(A) 10.5 ± 0.99<br>(B) 10.4 ± 0.95	NR	Acupressure	Placebo<br>(five acupuncture points that have not been effective in obesity treatment were selected)	8 wk / none	1. BMI<br>2. Body weight<br>3. WC<br>4. HC<br>5. Body fat ratio<br>6. Body fat mass (kg)<br>7. Skeletal muscle mass (kg)<br>8. Height	None
Huang 2004	60(30:30)	3~6	dual effulgence of Spleen and Stomach (30 cases),<br>Liver depression and qi stagnation (20 cases),<br>Spleen deficiency and dampness exuberance (10 cases)	Chuna + Acupressure	Non-medical management	3 mon / none	1. Body weight<br>2. Height<br>3. TER	NR
Huang 2021	120(60:60)	(A) 7.98 ± 1.03<br>(B) 7.95 ± 1.12	NR	HM	Non-medical management	12 wk / none	1. TER	NR

Lei 2006	160(130:30)	6~12	Spleen deficiency with dampness stagnation, Stomach fire effulgence	AT	Non-medical management	30 d / none	1. BMI<br>2. TER	NR
Li 2003	60(30:30)	(A) 12 ± 3<br>(B) 11 ± 3	NR	Chuna	Non-medical management	30 d / 6 mon	1. BMI<br>2. Body weight	NR
Li 2006	90(30:30:30 →85(26:29:30)	(A1) 16 ± 1.38<br>(A2) 15 ± 2.04<br>(B) 16 ± 1.95	NR	AT	Non-medical management	70 d / 30 d	1. BMI<br>2. Body weight<br>3. TER	NR
Li 2020	60(30:30)<br>→<br>56(27:29)	(A) 9.33 ± 1.59<br>(B) 9.24 ± 1.48	(A) Stomach heat with dampness obstruction (10 cases), Spleen deficiency with dampness obstruction (11 cases), Liver depression and qi stagnation (3 cases), dual deficiency of Spleen and Kidney (1 case), yin deficiency with internal heat (2 cases)<br>(B) Stomach heat with dampness obstruction (11 cases), Spleen deficiency with dampness obstruction (12 cases), Liver depression and qi stagnation (3 cases), dual deficiency of Spleen and Kidney (2 cases), yin deficiency with internal heat (1 case)	Chuna + AT	Non-medical management	6 wk / none	1. BMI<br>2. Body weight<br>3. WC<br>4. TER	None
Lin 2015	60(30:30)	(A) 13.70 ± 3.65<br>(B) 14.81 ± 3.24	Spleen deficiency with dampness obstruction	Cupping + Acupressure	Chuna	4 wk / none	1. BMI<br>2. Body weight<br>3. Body fat ratio<br>4. TER	None
Liu 2016	98(52:46)	(A) 10.34 ± 3.46<br>(B) 10.27 ± 3.54	NR	Chuna + Acupressure	Non-medical management	12 wk / none	1. BMI<br>2. Body weight<br>3. Height<br>4. TER	NR
Long 2019	95(47:48)	(A) 10.03 ± 2.15<br>(B) 9.82 ± 2.30	Spleen deficiency with dampness obstruction, Stomach heat with dampness obstruction, Liver depression and qi stagnation, dual deficiency of Spleen and Kidney, yin deficiency with internal heat	Chuna	Non-medical management	12 wk / none	1. BMI<br>2. Body weight<br>3. WC<br>4. TER	None

Pang 2010	72(37:35)	NR	Spleen deficiency with dampness obstruction, Stomach heat with dampness obstruction, dual deficiency of Spleen and Kidney, yin deficiency with internal heat	HM	Non-medical management	6 mon / none	1. BMI	NR
Qin 2016	64(32:32) →60(30:30)	(A) 11.03 ± 2.01<br>(B) 10.90 ± 2.14	phlegm-dampness internal exuberance	HM	Non-medical management	12 wk / 3 mon	1. BMI<br>2. TER	None
Shen 2001	87(45:42)	(A) 10.2<br>(B) 10.5	NR	HM	Fenfluramine	3 mon / none	1. TER	(A) None<br>(B) Four children forced to stop the drug due to obvious side effects, such as drowsiness affecting learning, mental depression, severe anorexia and so on. Another children have dry mouth, dizziness, fatigue, mild diarrhea and so on.
Song 2017	72(36:36) →62(31:31)	5~13	Spleen-Stomach dampness-heat	Chuna	HM	5 wk / none	1. BMI<br>2. Body weight<br>3. WHR<br>4. TER	NR
Tai 2006	68(30:18:20)	6~14	Spleen deficiency with dampness obstruction	Chuna	Non-medical management	3 mon / none	1. BMI<br>2. Body weight<br>3. WC<br>4. HC<br>5. WHR<br>6. Body fat ratio<br>7. Height<br>8. TER	A transient increase in appetite (1 case, not specified which of the two groups)
Tang 2016	100(50:50)	(A) 9.2 ± 1.0<br>(B) 9.6 ± 1.1	NR	Moxibustion	Non-medical management	3 mon / none	1. Body weight<br>2. Height	NR

Wang 2019a	64(32:32) →60(30:30)	7~13	Stomach heat with dampness obstruction	Cupping	Non-medical management	4 wk / none	1. BMI<br>2. Body weight<br>3. WC<br>4. HC<br>5. WHR<br>6. Body fat ratio<br>7. TER	(A) None<br>(B) NR
Wang 2019b	97(49:48)	(A) 8.73 ± 6.19<br>(B) 8.71 ± 6.12	NR	HM	Non-medical management	3 mon / none	1. TER	NR
Wang 2021a	74(37:37)	(A) 7.61 ± 2.47<br>(B) 7.54 ± 2.39	NR	HM	Non-medical management	1 mon / none	1. TER	NR
Wang 2021b	80(40:40)	(A) 6.34 ± 3.67<br>(B) 6.88 ± 3.56	NR	Chuna	Non-medical management	NR / NR	1. BMI<br>2. Body fat ratio	NR
Xiao 2008	99(50:49)	(A) 8 ± 2.5<br>(B) 7 ± 2.6	phlegm turbidity obstruction, blood stasis due to qi stagnation	HM	HM	3 mon / none	1. BMI<br>2. TER	NR
Xing 2009	139(88:51)	10~14	food accumulation, heavy dampness, Spleen qi deficiency	HM + AT	AT	2 mon / none	1. BMI	NR
Xiong 2014	80(40:40)	(A) 9.37 ± 0.59<br>(B) 9.29 ± 0.46	NR	HM + AT	Non-medical management	20 d / none	1. BMI	NR
Yang 2003	55(35:20)	(A) 10.23<br>(B) 10	Spleen deficiency with dampness obstruction	HM	Non-medical management	3 mon / none	1. Body weight<br>2. Height<br>3. TER	No other adverse reactions were found except that the stool texture became thinner, the odor was obvious, and the stool frequency increased to twice a day.
Yang 2018	108(54:54)	(A) 6~14<br>(B) 7~14	NR	HM	Non-medical management	12 wk / 6 mon	1. BMI<br>2. Body weight<br>3. Body fat ratio<br>4. TER	N.S<br>(A) mild rash (1 case)<br>(B) none
Yao 2019	60(30:30)	7~14	Spleen deficiency with phlegm obstruction	HM + Acupressure	Non-medical management	3 mon / none	1. BMI<br>2. TER	None

Yu 1998	253(101:101: 51)	(A1) 10.5<br>(A2) 10.2<br>(B) 10.4	NR	(A1) Moxibustion<br>(A2) Acupressure	Non-medical management	3 mon / none	1. Body weight<br>2. Height	NR
Zhang 2015	60(30:30)	5~12	NR	Chuna	Non-medical management	NR / NR	1. BMI<br>2. Body fat ratio<br>3. TER	NR
Zhang 2020	100(50:50)	(A) 6.53 ± 1.58<br>(B) 6.29 ± 1.62	NR	HM + AT	AT	2 mon / none	1. BMI<br>2. TER	NR
Zhou 2016	60(31:29)	12.75 ± 1.45	excess pattern	HM	Non-medical management	3 mon / none	1. BMI<br>2. WC<br>3. TER	NR
Zhu 2000	45(25:20)	4~6	gastrointestinal excess heat, Liver depression and qi stagnation, Spleen deficiency with dampness obstruction	Chuna + Acupressure	Chuna	12 wk / none	1. Body weight<br>2. Height<br>3. TER	NR

AT, acupuncture; BMI, body mass index; F/U, follow-up; HC, hip circumference; HM, herbal medicine; NR, not recorded; N.S, not significant between the groups; SD, standard deviation; TER, total effective rate; WC, waist circumference; WHR, waist-hip ratio.
