# Supplementary material for: Comparative Effectiveness of East Asian Traditional Medicine for Childhood Simple Obesity: A Systematic Review and Network Meta-Analysis
Source: Int J Environ Res Public Health. 2022 Oct 11;19(20):12994. doi: 10.3390/ijerph192012994 (PMC9602315; doi:10.3390/ijerph192012994)
Supplement: Supplementary file 1 [file ijerph-19-12994-s001.zip › Supplement S4.pdf]

Supplement S4. Details of herbal medicine used in the included studies

Study ID	Name of herbal medicine	Dosage form	Administration duration	Composition and dose of individual herb	Modifying components	Manufacturing company (Chinese patent medicine)
Huang 2021	modified Wendan-tang	Decoction	12 weeks	Alismatis Rhizoma, Phyllostachyos Caulis in Taeniam, Pinelliae Tuber, Polygalae Radix, Citri Unshius Pericarpium, Acori Graminei Rhizoma each 10 g, Glycyrrhizae Radix et Rhizoma 6 g, Zingiberis Rhizoma Recens 5 pieces, Zizyphi Fructus 3 g<br>(per day)	-edema: Benincasae Exocarpium 30 g, Polyporus 10 g<br>-anorexia: Atractylodis Rhizoma Alba, Codonopsis Pilosulae Radix each 10 g<br>(per day)	Not applicable
Pang 2010	-spleen deficiency with dampness obstruction: modified Pingwei-san<br>-stomach heat with dampness obstruction: modified Xiehuang-san<br>-dual deficiency of spleen and kidney: modified Liujunzi-tang<br>-yin deficiency with internal heat: modified Qiju-Dihuang-hwan	Not recorded	60 days	Not recorded	None	Not applicable
Qin 2016	modified Cangfu-Daotan-granule	Granule	12 weeks	Atractylodis Rhizoma, Cyperi Rhizoma, Scutellariae Radix, Poria Sclerotium, Curcumae Radix, Trichosanthis Semen, Angelicae Gigantis Radix each 10 g, Pinelliae Tuber, Citri Unshius Pericarpium, Ponciri Fructus Immaturus each 6 g, Amomi Fructus Rotundus, Magnoliae Cortex, Coptidis Rhizoma each 3 g<br>(per 2/3~2 days according to the age)	None	Jiangfen Jiangyin Tianjiang Pharmaceutical Co., Ltd.
Shen 2001	Jiangzhi-tablet	Tablet	3 months	Rhei Radix et Rhizoma 6~15 g<br>(per day)	None	Not applicable
Song 2017	Fangfeng-tongsheng-pill	Pill	30 days	Saposhnikoviae Radix, Schizonepetae Spica, Menthae Herba, Ephedrae Herba, Rhei Radix et Rhizoma, Natrii Sulfas, Gardeniae Fructus, Talcum, Platycodonis Radix, Gypsum Fibrosum, Cnidii Rhizoma, Angelicae Gigantis Radix, Paeoniae Radix, Scutellariae Radix, Forsythiae Fructus, Glycyrrhizae Radix et Rhizoma, Atractylodis Rhizoma Alba	None	Beijing Tongrentang Technology Development Co., Ltd.
Wang 2019b	Yiqi-Jianpi-tang	Decoction	3 months	Stigma Maydis 15 g, Astragali Radix, Crataegi Fructus each 12 g, Pseudostellariae Radix 9 g, Citri Unshius Pericarpium, Salviae Miltiorrhizae Radix, Pinelliae Tuber each 6 g<br>(per 1~1.5 days according to the age)	None	Not applicable
Wang 2021a	Yiqi-Jianpi-tang	Decoction	1 month	Stigma Maydis 15 g, Astragali Radix, Crataegi Fructus each 12 g, Pseudostellariae Radix 9 g, Citri Unshius Pericarpium, Salviae Miltiorrhizae Radix, Pinelliae Tuber each 6 g<br>(per 1~1.5 days according to the age)	None	Not applicable

Xiao 2008	(A) Jianfei oral liquid<br>(B) Fangfeng-tongsheng-pill	(A) Oral liquid<br>(B) Pill	3 months	(A) Cassiae Semen, Coicis Semen each 15 g, Poria Sclerotium 12 g, Pharbitidis Semen, Nelumbinis Folium, Atractylodis Rhizoma, Atractylodis Rhizoma Alba, Citri Unshius Pericarpium, Salviae Miltiorrhizae Radix, Crataegi Fructus each 10 g, Pinelliae Tuber, Arecae Pericarpium each 8 g (per 1/6~1/3 days according to the age)<br>(B) Not recorded	None	Not applicable
Xing 2009	Erchen-tang	Decoction	2 months	Poria Sclerotium 10 g, Mume Fructus 9 g, Pinelliae Tuber, Citri Unshius Pericarpium each 7 g, Zingiberis Rhizoma Recens, Glycyrrhizae Radix et Rhizoma each 6 g<br>(per 2 days)	-food accumulation: Crataegi Fructus 9 g<br>-heavy dampness: Polygoni Cuspidati Rhizoma et Radix 7 g<br>-spleen qi deficiency: Codonopsis Pilosulae Radix 10 g<br>(per 2 days)	Not applicable
Xiong 2014	Erchen-tang	Decoction	20 days	Poria Sclerotium 10 g, Mume Fructus 9 g, Pinelliae Tuber, Citri Unshius Pericarpium each 7 g, Zingiberis Rhizoma Recens, Glycyrrhizae Radix et Rhizoma each 6 g<br>(per day)	None	Not applicable
Yang 2003	Children's Jianfei-capsule	Capsule	3 months	Pinelliae Tuber, Poria Sclerotium, Alismatis Rhizoma, Magnoliae Cortex	None	Yunnan University of Traditional Chinese Medicine Pharmaceutical Factory
Yang 2018	modified Wendan-tang	Decoction	12 weeks	Poria Sclerotium 15 g, Alismatis Rhizoma, Phyllostachyos Caulis in Taeniam, Pinelliae Tuber, Polygalae Radix, Acori Graminei Rhizoma, Citri Unshius Pericarpium each 10 g, Glycyrrhizae Radix et Rhizoma 6 g, Zizyphi Fructus 3 g, Zingiberis Rhizoma Recens 5 pieces<br>(per day)	None	Not applicable
Yao 2019	modified Erchen-tang	Decoction	3 months	Poria Sclerotium, Pinelliae Tuber, Salviae Miltiorrhizae Radix, Nelumbinis Folium, Ponciri Fructus Immaturus, Arecae Semen, Crataegi Fructus each 9 g, Citri Unshius Pericarpium 6 g, Glycyrrhizae Radix et Rhizoma 3 g<br>(per day)	None	Not applicable
Zhang 2020	Erchen-tang	Decoction	2 months	Poria Sclerotium 10 g, Mume Fructus 9 g, Pinelliae Tuber, Citri Unshius Pericarpium each 7 g, Zingiberis Rhizoma Recens, Glycyrrhizae Radix et Rhizoma each 6 g<br>(per day)	-food accumulation: Crataegi Fructus 9 g<br>-heavy dampness: Polygoni Cuspidati Rhizoma et Radix 7 g<br>-spleen qi deficiency: Codonopsis Pilosulae Radix 10 g<br>(per day)	Not applicable

Zhou 2016	Fangfeng-tongsheng-san	Decoction	3 months	Saposhnikoviae Radix, Schizonepetae Spica, Forsythiae Fructus, Menthae Herba, Cnidii Rhizoma, Angelicae Gigantis Radix, Paeoniae Radix, Atractylodis Rhizoma Alba, Gardeniae Fructus, Rhei Radix et Rhizoma, Natrii Sulfas, Gypsum Fibrosum, Scutellariae Radix, Platycodonis Radix, Glycyrrhizae Radix et Rhizoma, Talcum	None	Not applicable
