# Supplementary material for: Comparative Effectiveness of East Asian Traditional Medicine for Childhood Simple Obesity: A Systematic Review and Network Meta-Analysis
Source: Int J Environ Res Public Health. 2022 Oct 11;19(20):12994. doi: 10.3390/ijerph192012994 (PMC9602315; doi:10.3390/ijerph192012994)
Supplement: Supplementary file 1 [file ijerph-19-12994-s001.zip › Supplement S5.pdf]

Supplement S5. Details of non-pharmacological EATMs used in the included studies

Study ID	Stimulation method	Acupoint	Needle retention or acupressure time	Frequency	Treatment duration	Total sessions	Others
Cao 2021	Auricular acupressure	Shenmen, Endocrine, Sympathetic, Lower Tragus, Sanjiao, Subcortex<br>-1-2 adjunct points selected based on the pattern identification<br>*dampness stagnation due to spleen deficiency: Spleen, Stomach, Lung, Heart<br>*stomach heat and dampness stagnation: Stomach, Liver, Large Intestine, Small Intestine<br>*deficiency of spleen and kidney: Spleen, Kidney, Bladder	2-3 min/time (better to produce slight pain)	4 times a day	3 months	360	Use sterile auricular point plasters. Plasters were replaced every other day
Cha 2019	Auricular acupressure	Hunger, Endocrine, Spleen, Shenmen, Stomach	5 min/time (Participants were instructed to slightly press the seed-applied areas before meals and whenever they felt hungry)	once a week	8 weeks	8	Use Vaccaria seeds as materials
Cha 2020	Auricular acupressure	Hunger, Endocrine, Spleen, Shenmen, Stomach	5 min/time (Participants were instructed to slightly press the seed-applied areas before meals and whenever they felt hungry)	once a week	8 weeks	8	Use ear pellets (seeds) as materials
Huang 2004	Chuna	-push down from the child's vertebral column along both sides of the spine (5-7 times)<br>-massage the bilateral BL23, BL20 (50 times), abdomen (100 times)<br>-push from the xiphoid process of the child along the two ribs (50 times)<br>-massage bilateral BL57 to the heel (100 times)<br>*dual effulgence of spleen and stomach: clear the large intestine meridian, relax six bowels, clear the stomach meridian (100 times), massage ST25, LI11, SP6, LI4 (50 times)<br>*liver depression and qi stagnation: clear the liver meridian, massage GB21, LV3, SP6 (50 times)<br>*spleen deficiency and dampness exuberance: transport spleen, transport bagua, massage ST25, ST37, SP4, ST36 (50 times)	Not recorded	once a day	3 months	90	None
	Auricular acupressure	*dual effulgence of spleen and stomach: Hunger, Large Intestine, Small Intestine, Stomach, Heart, Sympathetic<br>*liver depression and qi stagnation: Mouth, Liver, Gallbladder, Shenmen, Subcortex, Endocrine<br>*spleen deficiency and dampness exuberance: Spleen, Hunger, Stomach, Bladder, Kidney, Sanjiao, Lung, Subcortex	5 min	3-5 times a day	3 months	270~450	Use magnetic beads as materials

Lei 2006	Manual acupuncture	*spleen deficiency with dampness stagnation: CV12, SP3, BL20, SP6, ST36, CV4, CV6<br>*stomach fire effulgence: ST44, LI11, LI4, ST37, ST25, TW6	Not recorded	Not recorded	30 days	Not recorded	*spleen deficiency with dampness stagnation: slight twirling lifting-thrusting supplementation method<br>*stomach fire effulgence: substantial twirling lifting-thrusting draining method
Li 2003	Chuna	Jiaji acupoints on both sides of the governor vessel in the back and the acupoints on the stomach meridian of Foot-Yangming, BL21, BL20, BL23, CV13, CV12, CV10	30 min	once a day	30 days	30	None
Li 2006	Electroacupuncture	(A1) SP6, ST25, ST36, LI11, ST40, ST44, CV12, BL20, BL23, CV6, SP9, ST37, LV3<br>(A2) Hunger, Subcortex, Shenmen, Adrenal gland, Sanjiao, Spleen, Stomach, Lung, Mouth, Large intestine, Lower rectum	(A1) 10 min<br>(A2) 15-20 min	(A1) once a day<br>(A2) once a day	70 days	(A1) 60<br>(A2) 60	(A1) 0.32×65mm (Huatuo); deqi; G6805 electroacupuncture therapeutic apparatus, continuous wave, current intensity 3mA, continuous frequency 7Hz<br>(A2) 0.32×25mm (Huatuo); deqi; G6805 electroacupuncture device, continuous wave, the intensity should be tolerated by the patient
Li 2020	Chuna	CV12, CV8, ST25, CV6, CV4, CV3, BL20, BL21, BL18, BL25, BL23, LI14, LI11, BL36, GB31, BL40, BL57, ST34, ST36, ST40<br>*stomach heat with dampness obstruction: ST44, LI11, ST37<br>*spleen deficiency with dampness obstruction: CV12, ST40, SP9<br>*liver depression and qi stagnation: LV3, LV14, LI4, flank<br>*dual deficiency of spleen and kidney: BL23, GV4, KI3, governor vessel<br>*yin deficiency with internal heat: KI3, ST44, inner calf	Not recorded	once a day	6 weeks	30	None
	Manual acupuncture	CV12, CV9, CV4, ST25, SP15, LI11, TW6, ST44, ST40, ST37, SP6, SP9<br>*stomach heat with dampness obstruction: LI4<br>*spleen deficiency with dampness obstruction: PC6, ST36<br>*liver depression and qi stagnation: LV14, LV3<br>*dual deficiency of spleen and kidney: CV6, BL20, BL23, ST36<br>*yin deficiency with internal heat: KI3, LU7	30 min	once a day	6 weeks	30	0.20×25mm (Huatuo);<br>deqi
Lin 2015	Fire cupping	CV12, CV4, bilateral ST25, bilateral ST28, bilateral ST36, bilateral SP9	5 min	once a day	4 weeks	24	None
	Acupressure	CV12, CV4, bilateral ST25, bilateral ST28, bilateral ST36, bilateral SP9	2 hr	once a day	4 weeks	24	Use Manji magnetic stickers as materials
	Chuna	massage the abdomen, CV12, CV4, bilateral ST25, bilateral ST28, bilateral ST36, bilateral SP9	6 min (abdomen), 1 min/acupoint	once a day	4 weeks	24	None
Liu 2016	Chuna	rub the spleen meridian, stomach meridian, bladder meridian (5-10 times), massage CV12, ST25, CV4, CV6, ST32, GB30, ST36, BL57, SP6 (1-2 min)	30-40 min	once a day	12 weeks	84	None
	Auricular acupressure	Spleen, Stomach, Esophagus, Endocrine, Hunger, Sympathetic	10-20 times/acupoint	3-5 times/day	12 weeks	252-420	Use Vaccaria seeds as materials

Long 2019	Chuna	*spleen deficiency with dampness obstruction: supplement the spleen meridian (3-10 min), clear the liver meridian (2-5 min), rub banmen, neibagua (1-3 min), massage the abdomen (2-5 min), spine pinching (8-10 times)<br>*stomach heat with dampness obstruction: supplement the spleen meridian (3-10 min), clear the stomach and large intestine meridian (2-5 min), massage bilateral ST40, SP9 (1 min)<br>*liver depression and qi stagnation: supplement the spleen meridian (3-10 min) clear the liver meridian (2-5 min), transport neibagua (1 min), rub flank (1 min), massage bilateral LV3, GB34 (1 min)<br>*dual deficiency of spleen and kidney: supplement the spleen meridian (3-10 min), supplement the kidney meridian (2-5 min), massage BL23, GV4 (1 min), spine pinching (8-10 times)<br>*yin deficiency with internal heat: supplement the spleen meridian (3-10 min), clear the liver meridian (2-5 min), pinch and rub errenshangmaxue (3-10 min), massage bilateral KI3, SP6 (1 min)	Not recorded	once a day	12 weeks	60	None
Song 2017	Chuna	push the spleen, rub banmen, massage the abdomen, spine pinching, massage ST36	Not recorded	once a day	5 weeks	30	None
Tai 2006	Chuna	-supplement the spleen meridian (300 times), clear the stomach meridian (100 times), clear the large intestine meridian (200 times), rub banmen (100 times), clear the small intestine meridian (100 times), transport neibagua (50 times)<br>-massage CV21 (10 times), abdomen (3-5 min), CV12 (2 min), ST25 (100 times), CV9, CV6, ST25, ST24, ST26 (30 min)<br>-spine pinching (3-5 times), massage BL20, BL21, ST36 (30 min), CV15 (100 times), push upward the lumbosacral region (100 times), rub the lumbosacral area (to the degree of mild hyperemia of the local skin)	40 min	once every other day	3 months	45	(A1) Use weight loss massage cream<br>(A2) Use Johnson & Johnson baby oil
Tang 2016	Light-moxibustion	CV14, Endocrine, ST25, CV6, ST40, SP6, CV12, ST36, Sympathetic, SP15, CV4, SP9<br>*3-5 acupoints each time	2-3 min/acupoint	once a day	3 months	90	Use a light moxibustion weight loss instrument, the wavelength is set to 400~950nm, the illumination is 18Lx, the external temperature is controlled at 40°C. The power of the light moxibustion machine is 170W.
Wang 2019a	Cupping therapy	ST25, ST36, CV12, ST40, CV4, SP15, LI11, BL20, ST34, ST28, BL21, push downward the lumbosacral region	5-10 min	5 days a week	4 weeks	20	According to the treatment site, three methods of cupping were selected: flash cupping method, slide cupping method and retained cupping method
Wang 2021b	Chuna	massage the abdomen, lift the skin, massage the thighs, massage and tap the buttocks, shoulders and back, massage ST36 with the thumb	Not recorded	Not recorded	Not recorded	Not recorded	None

Xing 2009	Electroacupuncture	CV6, CV12, ST28, ST25, SP15<br>*food accumulation: ST37<br>*heavy dampness: SP9<br>*spleen qi deficiency: ST36	15 min	once every other day	2 months	30	1.5 inch 32 gauge needles (Huatuo); deqi; D6805-II electroacupuncture therapeutic apparatus (sparse and dense waves)
Xiong 2014	Electroacupuncture	CV6, CV12, ST28, ST25, SP15	15 min	once every other day	20 days	10	1.5 inch 32 gauge needles (Huatuo); deqi; D6805-II electroacupuncture therapeutic apparatus
Yao 2019	Auricular acupressure	Hunger, Brain, Endocrine, Spleen, Large intestine	Not recorded	Compress several times a day	3 months	Not recorded	None
Yu 1998	Light-moxibustion	CV12, CV14, bilateral ST36, ST40, SP9, SP6, Endocrine, Sympathetic, ST25, SP15, CV6, CV4<br>*3-5 acupoints each time	2-3 min/acupoint	once a day	3 months	90	The wavelength is 400 ~ 950nm, the illumination is 18Lx
	Auricular acupressure	Shenmen, Endocrine, Sympathetic, Adrenal gland, Stomach, Spleen<br>*3-4 acupoints each time	3-5 min/acupoint	3 times a day	3 months	270	Use Vaccaria seeds as materials
Zhang 2015	Chuna	massage the abdomen, lift the skin, massage the thighs, and massage and tap the buttocks, shoulders and back	Not recorded	Not recorded	Not recorded	Not recorded	None
Zhang 2020	Electroacupuncture	CV6, CV12, ST28, ST25, SP15<br>*food accumulation: ST37, ST39<br>*heavy dampness: SP9<br>*spleen qi deficiency: ST36	15 min	once every other day	2 months	30	1.5 inch 32 gauge filiform needles (Huatuo); deqi; XS-998B06 electroacupuncture therapeutic apparatus (Nanjing Komatsu Medical Instrument Research Institute, sparse and dense waves)
Zhu 2000	Auricular acupressure	*gastrointestinal excess heat: Hunger, Large intestine, Small intestine, Stomach, Constipation, Sympathetic, Heart<br>*liver depression and qi stagnation: Mouth, Liver, Gallbladder, Shenmen, Subcortex, Endocrine, Ovary<br>*spleen deficiency with dampness obstruction: Spleen, Stomach, Bladder, Kidney, Sanjiao, Lung, Subcortex	5 min	3-5 times/day	12 weeks	252-420	None
	Chuna	Push the ridge (5-7 times), massage the abdomen (100 times), massage BL57 (100 times)<br>*gastrointestinal excess heat: clear the large intestine meridian, remove six bowels, clear the stomach meridian (100 times)<br>*liver depression and qi stagnation: clear the liver meridian, massage GB21 (50 times)<br>*spleen deficiency with dampness obstruction: transport spleen, transport bagua, massage ST36 (50 times)	Not recorded	once a day	12 weeks	84	None
