# Supplementary material for: Comparative Effectiveness of East Asian Traditional Medicine for Childhood Simple Obesity: A Systematic Review and Network Meta-Analysis
Source: Int J Environ Res Public Health. 2022 Oct 11;19(20):12994. doi: 10.3390/ijerph192012994 (PMC9602315; doi:10.3390/ijerph192012994)
Supplement: Supplementary file 1 [file ijerph-19-12994-s001.zip › Supplement S6.pdf]

Supplement S6. League table for pairwise meta-analysis (right upper part) and network meta-analysis (left lower part) effect estimates: height

**Acupressure**	-	-	-	-1.70 (-4.14,0.74)	**8.50 (5.50,11.50)**	1.53 (-2.65,5.71)
**8.16 (0.74,15.58)**	**Chuna**	-0.70 (-5.99, 4.59)	-	-	1.64 (-4.14,7.42)	-
**9.63 (2.99,16.27)**	1.47 (-3.95,6.89)	**Chuna + acupressure**	-	-	-3.61 (-7.27,0.04)	-
4.66 (-4.76,14.08)	-3.50 (-13.23,6.24)	-4.96 (-14.12,4.19)	**HM**	-	2.72 (-3.96,9.40)	-
-0.80 (-5.60,4.00)	**-8.96 (-15.73,-2.18)**	**-10.43 (-16.34,-4.51)**	-5.46 (-14.37,3.44)	**Moxa**	**8.16 (3.95,12.37)**	-
**7.38 (2.43,12.33)**	-0.78 (-6.31,4.76)	-2.24 (-6.68,2.19)	2.72 (-5.30,10.74)	**8.18 (4.30,12.06)**	**None**	-
1.53 (-4.56,7.62)	-6.63 (-16.23,2.97)	-8.10 (-17.10,0.91)	-3.13 (-14.35,8.08)	2.33 (-5.42,10.08)	-5.85 (-13.70,1.99)	**Placebo**

Results are presented as the mean difference (95% confidence interval). The comparison must be read from left to right. A mean difference higher than zero indicates that treatment on the left is favored in both pairwise and network meta-analyses. Bold value means a significant difference between the groups.

HM, herbal medicine; Moxa, moxibustion; None, non-medical management.
