# Supplementary material for: Comparative Effectiveness of East Asian Traditional Medicine for Childhood Simple Obesity: A Systematic Review and Network Meta-Analysis
Source: Int J Environ Res Public Health. 2022 Oct 11;19(20):12994. doi: 10.3390/ijerph192012994 (PMC9602315; doi:10.3390/ijerph192012994)
Supplement: Supplementary file 1 [file ijerph-19-12994-s001.zip › Supplement S7.pdf]

Supplement S7. League table for pairwise meta-analysis (right upper part) and network meta-analysis (left lower part) effect estimates: total effective rate

**AT**	-	-	-	-	-	-	-	-	**0.74 (0.58,0.95)**	-	**2.60 (1.75,3.86)**
**2.12 (1.39,3.24)**	**Acupressure**	-	-	-	-	-	-	-	-	-	**1.23 (1.07,1.40)**
**2.32 (1.52,3.52)**	1.09 (0.90,1.33)	**Chuna**	-	0.85 (0.64,1.14)	-	1.00 (0.84,1.18)	-	1.13 (0.89,1.42)	-	-	1.19 (0.90,1.58)
1.40 (0.81,2.39)	**0.66 (0.44,0.97)**	**0.60 (0.41,0.89)**	**Chuna + AT**	-	-	-	-	-	-	-	**1.86 (1.30,2.67)**
**2.10 (1.37,3.22)**	0.99 (0.79,1.23)	0.91 (0.77,1.07)	**1.50 (1.01,2.24)**	**Chuna + acupressure**	-	-	-	-	-	-	3.77 (0.08,185.52)
**2.12 (1.32,3.39)**	1.00 (0.75,1.34)	0.91 (0.69,1.22)	1.52 (0.97,2.37)	1.01 (0.75,1.37)	**Cupping**	-	-	-	-	-	1.23 (0.96,1.57)
**2.32 (1.47,3.65)**	1.09 (0.84,1.42)	1.00 (0.84,1.20)	**1.66 (1.08,2.54)**	1.10 (0.86,1.41)	1.09 (0.78,1.53)	**Cupping + acupressure**	-	-	-	-	-
**2.19 (1.42,3.38)**	1.03 (0.82,1.30)	0.95 (0.78,1.15)	**1.57 (1.05,2.35)**	1.04 (0.83,1.31)	1.03 (0.76,1.41)	0.95 (0.72,1.24)	**Fenfluramine**	1.02 (0.88,1.17)	-	-	-
**2.23 (1.49,3.34)**	1.05 (0.89,1.24)	0.96 (0.85,1.09)	**1.60 (1.10,2.32)**	1.06 (0.90,1.25)	1.05 (0.81,1.37)	0.96 (0.77,1.20)	1.02 (0.87,1.19)	**HM**	-	-	**1.23 (1.14,1.32)**
**0.74 (0.57,0.95)**	**0.35 (0.21,0.57)**	**0.32 (0.20,0.52)**	**0.53 (0.29,0.96)**	**0.35 (0.21,0.58)**	**0.35 (0.20,0.60)**	**0.32 (0.19,0.54)**	**0.34 (0.20,0.56)**	**0.33 (0.21,0.53)**	**HM + AT**	-	-
1.14 (0.60,2.17)	**0.54 (0.32,0.91)**	**0.49 (0.29,0.83)**	0.82 (0.44,1.52)	**0.55 (0.32,0.92)**	**0.54 (0.31,0.95)**	**0.49 (0.29,0.85)**	**0.52 (0.31,0.89)**	**0.51 (0.31,0.85)**	1.55 (0.78,3.08)	**HM + acupressure**	**2.27 (1.38,3.74)**
**2.60 (1.75,3.87)**	**1.23 (1.06,1.42)**	1.12 (0.98,1.28)	**1.86 (1.29,2.68)**	**1.24 (1.05,1.46)**	1.23 (0.95,1.58)	1.12 (0.90,1.40)	1.19 (1.00,1.41)	**1.17 (1.08,1.26)**	**3.52 (2.20,5.65)**	**2.27 (1.38,3.75)**	**None**

Results are presented as the risk ratio (95% confidence interval). The comparison must be read from left to right. A risk ratio higher than one indicates that treatment on the left is favored in both pairwise and network meta-analyses. Bold value means a significant difference between the groups.

AT, acupuncture; HM, herbal medicine; None, non-medical management.
