# Supplementary material for: Comparative Effectiveness of East Asian Traditional Medicine for Childhood Simple Obesity: A Systematic Review and Network Meta-Analysis
Source: Int J Environ Res Public Health. 2022 Oct 11;19(20):12994. doi: 10.3390/ijerph192012994 (PMC9602315; doi:10.3390/ijerph192012994)
Supplement: Supplementary file 1 [file ijerph-19-12994-s001.zip › Supplement S8.pdf]

Supplement S8. Quality of evidence by the GRADE approach

(1) BMI

Comparison		Direct evidence (pairwise meta-analysis)	Indirect evidence	Network meta-analysis
AT	Acupressure	-	Low<br>Risk of bias (-1)<br>Imprecision (-1)	Low<br>Risk of bias (-1)<br>Imprecision (-1)
AT	Chuna	-	Moderate<br>Risk of bias (-1)	Moderate<br>Risk of bias (-1)
AT	Chuna + AT	-	Low<br>Risk of bias (-1)<br>Imprecision (-1)	Low<br>Risk of bias (-1)<br>Imprecision (-1)
AT	Chuna + acupressure	-	Low<br>Risk of bias (-1)<br>Imprecision (-1)	Low<br>Risk of bias (-1)<br>Imprecision (-1)
AT	Cupping	-	Low<br>Risk of bias (-1)<br>Imprecision (-1)	Low<br>Risk of bias (-1)<br>Imprecision (-1)
AT	Cupping + acupressure	-	Low<br>Risk of bias (-1)<br>Imprecision (-1)	Low<br>Risk of bias (-1)<br>Imprecision (-1)
AT	HM	-	Moderate<br>Risk of bias (-1)	Moderate<br>Risk of bias (-1)
AT	HM + AT	Moderate<br>Risk of bias (-1)	Low<br>Risk of bias (-1)<br>Imprecision (-1)	Moderate<br>Risk of bias (-1)
AT	HM + acupressure	-	Low<br>Risk of bias (-1)<br>Imprecision (-1)	Low<br>Risk of bias (-1)<br>Imprecision (-1)
AT	None	Moderate<br>Risk of bias (-1)	Low<br>Risk of bias (-1)<br>Imprecision (-1)	Moderate<br>Risk of bias (-1)
AT	Placebo	-	Low<br>Risk of bias (-1)<br>Imprecision (-1)	Low<br>Risk of bias (-1)<br>Imprecision (-1)
Acupressure	Chuna	-	Low<br>Risk of bias (-1)<br>Imprecision (-1)	Low<br>Risk of bias (-1)<br>Imprecision (-1)
Acupressure	Chuna + AT	-	Low<br>Risk of bias (-1)<br>Imprecision (-1)	Low<br>Risk of bias (-1)<br>Imprecision (-1)
Acupressure	Chuna + acupressure	-	Low<br>Risk of bias (-1)<br>Imprecision (-1)	Low<br>Risk of bias (-1)<br>Imprecision (-1)
Acupressure	Cupping	-	Low<br>Risk of bias (-1)<br>Imprecision (-1)	Low<br>Risk of bias (-1)<br>Imprecision (-1)
Acupressure	Cupping + acupressure	-	Moderate<br>Risk of bias (-1)	Moderate<br>Risk of bias (-1)

Acupressure	HM	-	Low<br>Risk of bias (-1)<br>Imprecision (-1)	Low<br>Risk of bias (-1)<br>Imprecision (-1)
Acupressure	HM + AT	-	Low<br>Risk of bias (-1)<br>Imprecision (-1)	Low<br>Risk of bias (-1)<br>Imprecision (-1)
Acupressure	HM + acupressure	-	Low<br>Risk of bias (-1)<br>Imprecision (-1)	Low<br>Risk of bias (-1)<br>Imprecision (-1)
Acupressure	None	Low<br>Risk of bias (-1)<br>Imprecision (-1)	-	Low<br>Risk of bias (-1)<br>Imprecision (-1)
Acupressure	Placebo	Low<br>Risk of bias (-1)<br>Imprecision (-1)	-	Low<br>Risk of bias (-1)<br>Imprecision (-1)
Chuna	Chuna + AT	-	Low<br>Risk of bias (-1)<br>Imprecision (-1)	Low<br>Risk of bias (-1)<br>Imprecision (-1)
Chuna	Chuna + acupressure	-	Low<br>Risk of bias (-1)<br>Imprecision (-1)	Low<br>Risk of bias (-1)<br>Imprecision (-1)
Chuna	Cupping	-	Low<br>Risk of bias (-1)<br>Imprecision (-1)	Low<br>Risk of bias (-1)<br>Imprecision (-1)
Chuna	Cupping + acupressure	Low<br>Risk of bias (-1)<br>Imprecision (-1)	-	Low<br>Risk of bias (-1)<br>Imprecision (-1)
Chuna	HM	Low<br>Risk of bias (-1)<br>Imprecision (-1)	Moderate<br>Risk of bias (-1)	Moderate<br>Risk of bias (-1)
Chuna	HM + AT	-	Low<br>Risk of bias (-1)<br>Imprecision (-1)	Low<br>Risk of bias (-1)<br>Imprecision (-1)
Chuna	HM + acupressure	-	Low<br>Risk of bias (-1)<br>Imprecision (-1)	Low<br>Risk of bias (-1)<br>Imprecision (-1)
Chuna	None	Moderate<br>Risk of bias (-1)	Low<br>Risk of bias (-1)<br>Imprecision (-1)	Moderate<br>Risk of bias (-1)
Chuna	Placebo	-	Moderate<br>Risk of bias (-1)	Moderate<br>Risk of bias (-1)
Chuna + AT	Chuna + acupressure	-	Low<br>Risk of bias (-1)<br>Imprecision (-1)	Low<br>Risk of bias (-1)<br>Imprecision (-1)
Chuna + AT	Cupping	-	Low<br>Risk of bias (-1)<br>Imprecision (-1)	Low<br>Risk of bias (-1)<br>Imprecision (-1)
Chuna + AT	Cupping + acupressure	-	Low<br>Risk of bias (-1)<br>Imprecision (-1)	Low<br>Risk of bias (-1)<br>Imprecision (-1)
Chuna + AT	HM	-	Low<br>Risk of bias (-1)<br>Imprecision (-1)	Low<br>Risk of bias (-1)<br>Imprecision (-1)

Chuna + AT	HM + AT	-	Low<br>Risk of bias (-1)<br>Imprecision (-1)	Low<br>Risk of bias (-1)<br>Imprecision (-1)
Chuna + AT	HM + acupressure	-	Low<br>Risk of bias (-1)<br>Imprecision (-1)	Low<br>Risk of bias (-1)<br>Imprecision (-1)
Chuna + AT	None	Low<br>Risk of bias (-1)<br>Imprecision (-1)	-	Low<br>Risk of bias (-1)<br>Imprecision (-1)
Chuna + AT	Placebo	-	Low<br>Risk of bias (-1)<br>Imprecision (-1)	Low<br>Risk of bias (-1)<br>Imprecision (-1)
Chuna + acupressure	Cupping	-	Low<br>Risk of bias (-1)<br>Imprecision (-1)	Low<br>Risk of bias (-1)<br>Imprecision (-1)
Chuna + acupressure	Cupping + acupressure	-	Low<br>Risk of bias (-1)<br>Imprecision (-1)	Low<br>Risk of bias (-1)<br>Imprecision (-1)
Chuna + acupressure	HM	-	Low<br>Risk of bias (-1)<br>Imprecision (-1)	Low<br>Risk of bias (-1)<br>Imprecision (-1)
Chuna + acupressure	HM + AT	-	Low<br>Risk of bias (-1)<br>Imprecision (-1)	Low<br>Risk of bias (-1)<br>Imprecision (-1)
Chuna + acupressure	HM + acupressure	-	Low<br>Risk of bias (-1)<br>Imprecision (-1)	Low<br>Risk of bias (-1)<br>Imprecision (-1)
Chuna + acupressure	None	Low<br>Risk of bias (-1)<br>Imprecision (-1)	-	Low<br>Risk of bias (-1)<br>Imprecision (-1)
Chuna + acupressure	Placebo	-	Low<br>Risk of bias (-1)<br>Imprecision (-1)	Low<br>Risk of bias (-1)<br>Imprecision (-1)
Cupping	Cupping + acupressure	-	Moderate<br>Risk of bias (-1)	Moderate<br>Risk of bias (-1)
Cupping	HM	-	Low<br>Risk of bias (-1)<br>Imprecision (-1)	Low<br>Risk of bias (-1)<br>Imprecision (-1)
Cupping	HM + AT	-	Low<br>Risk of bias (-1)<br>Imprecision (-1)	Low<br>Risk of bias (-1)<br>Imprecision (-1)
Cupping	HM + acupressure	-	Low<br>Risk of bias (-1)<br>Imprecision (-1)	Low<br>Risk of bias (-1)<br>Imprecision (-1)
Cupping	None	Low<br>Risk of bias (-1)<br>Imprecision (-1)	-	Low<br>Risk of bias (-1)<br>Imprecision (-1)
Cupping	Placebo	-	Low<br>Risk of bias (-1)<br>Imprecision (-1)	Low<br>Risk of bias (-1)<br>Imprecision (-1)
Cupping + acupressure	HM	-	Low<br>Risk of bias (-1)<br>Imprecision (-1)	Low<br>Risk of bias (-1)<br>Imprecision (-1)

Cupping + acupressure	HM + AT	-	Low<br>Risk of bias (-1)<br>Imprecision (-1)	Low<br>Risk of bias (-1)<br>Imprecision (-1)
Cupping + acupressure	HM + acupressure	-	Low<br>Risk of bias (-1)<br>Imprecision (-1)	Low<br>Risk of bias (-1)<br>Imprecision (-1)
Cupping + acupressure	None	-	Low<br>Risk of bias (-1)<br>Imprecision (-1)	Low<br>Risk of bias (-1)<br>Imprecision (-1)
Cupping + acupressure	Placebo	-	Moderate<br>Risk of bias (-1)	Moderate<br>Risk of bias (-1)
HM	HM + AT	-	Low<br>Risk of bias (-1)<br>Imprecision (-1)	Low<br>Risk of bias (-1)<br>Imprecision (-1)
HM	HM + acupressure	-	Low<br>Risk of bias (-1)<br>Imprecision (-1)	Low<br>Risk of bias (-1)<br>Imprecision (-1)
HM	None	Moderate<br>Risk of bias (-1)	Low<br>Risk of bias (-1)<br>Imprecision (-1)	Moderate<br>Risk of bias (-1)
HM	Placebo	-	Moderate<br>Risk of bias (-1)	Moderate<br>Risk of bias (-1)
HM + AT	HM + acupressure	-	Low<br>Risk of bias (-1)<br>Imprecision (-1)	Moderate<br>Risk of bias (-1)
HM + AT	None	Low<br>Risk of bias (-1)<br>Imprecision (-1)	Moderate<br>Risk of bias (-1)	Moderate<br>Risk of bias (-1)
HM + AT	Placebo	-	Low<br>Risk of bias (-1)<br>Imprecision (-1)	Low<br>Risk of bias (-1)<br>Imprecision (-1)
HM + acupressure	None	Low<br>Risk of bias (-1)<br>Imprecision (-1)	-	Low<br>Risk of bias (-1)<br>Imprecision (-1)
HM + acupressure	Placebo	-	Low<br>Risk of bias (-1)<br>Imprecision (-1)	Low<br>Risk of bias (-1)<br>Imprecision (-1)
None	Placebo	-	Low<br>Risk of bias (-1)<br>Imprecision (-1)	Low<br>Risk of bias (-1)<br>Imprecision (-1)

(2) Body weight

Comparison		Direct evidence (pairwise meta-analysis)	Indirect evidence	Network meta-analysis
AT	Acupressure	-	Low<br>Risk of bias (-1)<br>Imprecision (-1)	Low<br>Risk of bias (-1)<br>Imprecision (-1)
AT	Chuna	-	Low<br>Risk of bias (-1)<br>Imprecision (-1)	Low<br>Risk of bias (-1)<br>Imprecision (-1)
AT	Chuna + AT	-	Low<br>Risk of bias (-1)<br>Imprecision (-1)	Low<br>Risk of bias (-1)<br>Imprecision (-1)

AT	Chuna + acupressure	-	Low<br>Risk of bias (-1)<br>Imprecision (-1)	Low<br>Risk of bias (-1)<br>Imprecision (-1)
AT	Cupping	-	Low<br>Risk of bias (-1)<br>Imprecision (-1)	Low<br>Risk of bias (-1)<br>Imprecision (-1)
AT	Cupping + acupressure	-	Low<br>Risk of bias (-1)<br>Imprecision (-1)	Low<br>Risk of bias (-1)<br>Imprecision (-1)
AT	HM	-	Low<br>Risk of bias (-1)<br>Imprecision (-1)	Low<br>Risk of bias (-1)<br>Imprecision (-1)
AT	Moxa	-	Very low<br>Risk of bias (-1)<br>Inconsistency (-2)<br>Imprecision (-1)	Very low<br>Risk of bias (-1)<br>Inconsistency (-2)<br>Imprecision (-1)
AT	None	Low<br>Risk of bias (-1)<br>Imprecision (-1)	-	Low<br>Risk of bias (-1)<br>Imprecision (-1)
AT	Placebo	-	Low<br>Risk of bias (-1)<br>Imprecision (-1)	Low<br>Risk of bias (-1)<br>Imprecision (-1)
Acupressure	Chuna	-	Low<br>Risk of bias (-1)<br>Imprecision (-1)	Low<br>Risk of bias (-1)<br>Imprecision (-1)
Acupressure	Chuna + AT	-	Low<br>Risk of bias (-1)<br>Imprecision (-1)	Low<br>Risk of bias (-1)<br>Imprecision (-1)
Acupressure	Chuna + acupressure	-	Low<br>Risk of bias (-1)<br>Imprecision (-1)	Low<br>Risk of bias (-1)<br>Imprecision (-1)
Acupressure	Cupping	-	Low<br>Risk of bias (-1)<br>Imprecision (-1)	Low<br>Risk of bias (-1)<br>Imprecision (-1)
Acupressure	Cupping + acupressure	-	Moderate<br>Risk of bias (-1)	Moderate<br>Risk of bias (-1)
Acupressure	HM	-	Low<br>Risk of bias (-1)<br>Imprecision (-1)	Low<br>Risk of bias (-1)<br>Imprecision (-1)
Acupressure	Moxa	Low<br>Risk of bias (-1)<br>Imprecision (-1)	Very low<br>Risk of bias (-1)<br>Inconsistency (-2)<br>Imprecision (-1)	Low<br>Risk of bias (-1)<br>Imprecision (-1)
Acupressure	None	Low<br>Risk of bias (-1)<br>Imprecision (-1)	Very low<br>Risk of bias (-1)<br>Inconsistency (-2)<br>Imprecision (-1)	Low<br>Risk of bias (-1)<br>Imprecision (-1)
Acupressure	Placebo	Low<br>Risk of bias (-1)<br>Imprecision (-1)	-	Low<br>Risk of bias (-1)<br>Imprecision (-1)
Chuna	Chuna + AT	-	Low<br>Risk of bias (-1)<br>Imprecision (-1)	Low<br>Risk of bias (-1)<br>Imprecision (-1)
Chuna	Chuna + acupressure	Low<br>Risk of bias (-1)<br>Imprecision (-1)	Moderate<br>Risk of bias (-1)	Moderate<br>Risk of bias (-1)

Chuna	Cupping	-	Low<br>Risk of bias (-1)<br>Imprecision (-1)	Low<br>Risk of bias (-1)<br>Imprecision (-1)
Chuna	Cupping + acupressure	Low<br>Risk of bias (-1)<br>Imprecision (-1)	-	Low<br>Risk of bias (-1)<br>Imprecision (-1)
Chuna	HM	Low<br>Risk of bias (-1)<br>Imprecision (-1)	Moderate<br>Risk of bias (-1)	Moderate<br>Risk of bias (-1)
Chuna	Moxa	-	Very low<br>Risk of bias (-1)<br>Inconsistency (-2)<br>Imprecision (-1)	Very low<br>Risk of bias (-1)<br>Inconsistency (-2)<br>Imprecision (-1)
Chuna	None	Moderate<br>Risk of bias (-1)	Low<br>Risk of bias (-1)<br>Imprecision (-1)	Moderate<br>Risk of bias (-1)
Chuna	Placebo	-	Moderate<br>Risk of bias (-1)	Moderate<br>Risk of bias (-1)
Chuna + AT	Chuna + acupressure	-	Low<br>Risk of bias (-1)<br>Imprecision (-1)	Low<br>Risk of bias (-1)<br>Imprecision (-1)
Chuna + AT	Cupping	-	Low<br>Risk of bias (-1)<br>Imprecision (-1)	Low<br>Risk of bias (-1)<br>Imprecision (-1)
Chuna + AT	Cupping + acupressure	-	Low<br>Risk of bias (-1)<br>Imprecision (-1)	Low<br>Risk of bias (-1)<br>Imprecision (-1)
Chuna + AT	HM	-	Low<br>Risk of bias (-1)<br>Imprecision (-1)	Low<br>Risk of bias (-1)<br>Imprecision (-1)
Chuna + AT	Moxa	-	Very low<br>Risk of bias (-1)<br>Inconsistency (-2)<br>Imprecision (-1)	Very low<br>Risk of bias (-1)<br>Inconsistency (-2)<br>Imprecision (-1)
Chuna + AT	None	Low<br>Risk of bias (-1)<br>Imprecision (-1)	-	Low<br>Risk of bias (-1)<br>Imprecision (-1)
Chuna + AT	Placebo	-	Low<br>Risk of bias (-1)<br>Imprecision (-1)	Low<br>Risk of bias (-1)<br>Imprecision (-1)
Chuna + acupressure	Cupping	-	Low<br>Risk of bias (-1)<br>Imprecision (-1)	Low<br>Risk of bias (-1)<br>Imprecision (-1)
Chuna + acupressure	Cupping + acupressure	-	Low<br>Risk of bias (-1)<br>Imprecision (-1)	Low<br>Risk of bias (-1)<br>Imprecision (-1)
Chuna + acupressure	HM	-	Moderate<br>Risk of bias (-1)	Moderate<br>Risk of bias (-1)
Chuna + acupressure	Moxa	-	Very low<br>Risk of bias (-1)<br>Inconsistency (-2)<br>Imprecision (-1)	Very low<br>Risk of bias (-1)<br>Inconsistency (-2)<br>Imprecision (-1)
Chuna + acupressure	None	Moderate<br>Risk of bias (-1)	Low<br>Risk of bias (-1)<br>Imprecision (-1)	Moderate<br>Risk of bias (-1)

Chuna + acupressure	Placebo	-	Low<br>Risk of bias (-1)<br>Imprecision (-1)	Low<br>Risk of bias (-1)<br>Imprecision (-1)
Cupping	Cupping + acupressure	-	Low<br>Risk of bias (-1)<br>Imprecision (-1)	Low<br>Risk of bias (-1)<br>Imprecision (-1)
Cupping	HM	-	Low<br>Risk of bias (-1)<br>Imprecision (-1)	Low<br>Risk of bias (-1)<br>Imprecision (-1)
Cupping	Moxa	-	Very low<br>Risk of bias (-1)<br>Inconsistency (-2)<br>Imprecision (-1)	Very low<br>Risk of bias (-1)<br>Inconsistency (-2)<br>Imprecision (-1)
Cupping	None	Low<br>Risk of bias (-1)<br>Imprecision (-1)	-	Low<br>Risk of bias (-1)<br>Imprecision (-1)
Cupping	Placebo	-	Low<br>Risk of bias (-1)<br>Imprecision (-1)	Low<br>Risk of bias (-1)<br>Imprecision (-1)
Cupping + acupressure	HM	-	Low<br>Risk of bias (-1)<br>Imprecision (-1)	Low<br>Risk of bias (-1)<br>Imprecision (-1)
Cupping + acupressure	Moxa	-	Moderate<br>Risk of bias (-1)	Moderate<br>Risk of bias (-1)
Cupping + acupressure	None	-	Low<br>Risk of bias (-1)<br>Imprecision (-1)	Low<br>Risk of bias (-1)<br>Imprecision (-1)
Cupping + acupressure	Placebo	-	Moderate<br>Risk of bias (-1)	Moderate<br>Risk of bias (-1)
HM	Moxa	-	Very low<br>Risk of bias (-1)<br>Inconsistency (-2)<br>Imprecision (-1)	Very low<br>Risk of bias (-1)<br>Inconsistency (-2)<br>Imprecision (-1)
HM	None	Moderate<br>Risk of bias (-1)	Low<br>Risk of bias (-1)<br>Imprecision (-1)	Moderate<br>Risk of bias (-1)
HM	Placebo	-	Moderate<br>Risk of bias (-1)	Moderate<br>Risk of bias (-1)
Moxa	None	Very low<br>Risk of bias (-1)<br>Inconsistency (-2)<br>Imprecision (-1)	Low<br>Risk of bias (-1)<br>Imprecision (-1)	Low<br>Risk of bias (-1)<br>Imprecision (-1)
Moxa	Placebo	-	Low<br>Risk of bias (-1)<br>Imprecision (-1)	Low<br>Risk of bias (-1)<br>Imprecision (-1)
None	Placebo	-	Low<br>Risk of bias (-1)<br>Imprecision (-1)	Low<br>Risk of bias (-1)<br>Imprecision (-1)

(3) Height

Comparison		Direct evidence (pairwise meta-analysis)	Indirect evidence	Network meta-analysis
Acupressure	Chuna	-	Low<br>Risk of bias (-1)<br>Imprecision (-1)	Low<br>Risk of bias (-1)<br>Imprecision (-1)
Acupressure	Chuna + acupressure	-	Low<br>Risk of bias (-1)<br>Imprecision (-1)	Low<br>Risk of bias (-1)<br>Imprecision (-1)
Acupressure	HM	-	Low<br>Risk of bias (-1)<br>Imprecision (-1)	Low<br>Risk of bias (-1)<br>Imprecision (-1)
Acupressure	Moxa	Low<br>Risk of bias (-1)<br>Imprecision (-1)	Moderate<br>Risk of bias (-1)	Moderate<br>Risk of bias (-1)
Acupressure	None	Moderate<br>Risk of bias (-1)	Low<br>Risk of bias (-1)<br>Imprecision (-1)	Moderate<br>Risk of bias (-1)
Acupressure	Placebo	Low<br>Risk of bias (-1)<br>Imprecision (-1)	-	-
Chuna	Chuna + acupressure	Low<br>Risk of bias (-1)<br>Imprecision (-1)	Low<br>Risk of bias (-1)<br>Imprecision (-1)	Low<br>Risk of bias (-1)<br>Imprecision (-1)
Chuna	HM	-	Low<br>Risk of bias (-1)<br>Imprecision (-1)	Low<br>Risk of bias (-1)<br>Imprecision (-1)
Chuna	Moxa	-	Low<br>Risk of bias (-1)<br>Imprecision (-1)	Low<br>Risk of bias (-1)<br>Imprecision (-1)
Chuna	None	Low<br>Risk of bias (-1)<br>Imprecision (-1)	Low<br>Risk of bias (-1)<br>Imprecision (-1)	Low<br>Risk of bias (-1)<br>Imprecision (-1)
Chuna	Placebo	-	Low<br>Risk of bias (-1)<br>Imprecision (-1)	Low<br>Risk of bias (-1)<br>Imprecision (-1)
Chuna + acupressure	HM	-	Low<br>Risk of bias (-1)<br>Imprecision (-1)	Low<br>Risk of bias (-1)<br>Imprecision (-1)
Chuna + acupressure	Moxa	-	Low<br>Risk of bias (-1)<br>Imprecision (-1)	Low<br>Risk of bias (-1)<br>Imprecision (-1)
Chuna + acupressure	None	Low<br>Risk of bias (-1)<br>Imprecision (-1)	Low<br>Risk of bias (-1)<br>Imprecision (-1)	Low<br>Risk of bias (-1)<br>Imprecision (-1)
Chuna + acupressure	Placebo	-	Low<br>Risk of bias (-1)<br>Imprecision (-1)	Low<br>Risk of bias (-1)<br>Imprecision (-1)
HM	Moxa	-	Low<br>Risk of bias (-1)<br>Imprecision (-1)	Low<br>Risk of bias (-1)<br>Imprecision (-1)
HM	None	Low<br>Risk of bias (-1)<br>Imprecision (-1)	-	Low<br>Risk of bias (-1)<br>Imprecision (-1)

HM	Placebo	-	Low<br>Risk of bias (-1)<br>Imprecision (-1)	Low<br>Risk of bias (-1)<br>Imprecision (-1)
Moxa	None	Moderate<br>Risk of bias (-1)	Low<br>Risk of bias (-1)<br>Imprecision (-1)	Moderate<br>Risk of bias (-1)
Moxa	Placebo	-	Low<br>Risk of bias (-1)<br>Imprecision (-1)	Low<br>Risk of bias (-1)<br>Imprecision (-1)
None	Placebo	-	Low<br>Risk of bias (-1)<br>Imprecision (-1)	Low<br>Risk of bias (-1)<br>Imprecision (-1)

(4) Total effective rate

Comparison		Direct evidence (pairwise meta-analysis)	Indirect evidence	Network meta-analysis
AT	Acupressure	-	Moderate<br>Risk of bias (-1)	Moderate<br>Risk of bias (-1)
AT	Chuna	-	Low<br>Risk of bias (-1)<br>Imprecision (-1)	Low<br>Risk of bias (-1)<br>Imprecision (-1)
AT	Chuna + AT	-	Low<br>Risk of bias (-1)<br>Imprecision (-1)	Low<br>Risk of bias (-1)<br>Imprecision (-1)
AT	Chuna + acupressure	-	Low<br>Risk of bias (-1)<br>Imprecision (-1)	Low<br>Risk of bias (-1)<br>Imprecision (-1)
AT	Cupping	-	Low<br>Risk of bias (-1)<br>Imprecision (-1)	Low<br>Risk of bias (-1)<br>Imprecision (-1)
AT	Cupping + acupressure	-	Moderate<br>Risk of bias (-1)	Moderate<br>Risk of bias (-1)
AT	Fenfluramine	-	Moderate<br>Risk of bias (-1)	Moderate<br>Risk of bias (-1)
AT	HM	-	Moderate<br>Risk of bias (-1)	Moderate<br>Risk of bias (-1)
AT	HM + AT	Low<br>Risk of bias (-1)<br>Imprecision (-1)	-	Low<br>Risk of bias (-1)<br>Imprecision (-1)
AT	HM + acupressure	-	Low<br>Risk of bias (-1)<br>Imprecision (-1)	Low<br>Risk of bias (-1)<br>Imprecision (-1)
AT	None	Moderate<br>Risk of bias (-1)	-	Moderate<br>Risk of bias (-1)
Acupressure	Chuna	-	Low<br>Risk of bias (-1)<br>Imprecision (-1)	Low<br>Risk of bias (-1)<br>Imprecision (-1)

Acupressure	Chuna + AT	-	Low<br>Risk of bias (-1)<br>Imprecision (-1)	Low<br>Risk of bias (-1)<br>Imprecision (-1)
Acupressure	Chuna +<br>acupressure	-	Low<br>Risk of bias (-1)<br>Imprecision (-1)	Low<br>Risk of bias (-1)<br>Imprecision (-1)
Acupressure	Cupping	-	Low<br>Risk of bias (-1)<br>Imprecision (-1)	Low<br>Risk of bias (-1)<br>Imprecision (-1)
Acupressure	Cupping +<br>acupressure	-	Low<br>Risk of bias (-1)<br>Imprecision (-1)	Low<br>Risk of bias (-1)<br>Imprecision (-1)
Acupressure	Fenfluramine	-	Low<br>Risk of bias (-1)<br>Imprecision (-1)	Low<br>Risk of bias (-1)<br>Imprecision (-1)
Acupressure	HM	-	Moderate<br>Risk of bias (-1)	Moderate<br>Risk of bias (-1)
Acupressure	HM + AT	-	Moderate<br>Risk of bias (-1)	Moderate<br>Risk of bias (-1)
Acupressure	HM + acupressure	-	Low<br>Risk of bias (-1)<br>Imprecision (-1)	Low<br>Risk of bias (-1)<br>Imprecision (-1)
Acupressure	None	Moderate<br>Risk of bias (-1)	-	Moderate<br>Risk of bias (-1)
Chuna	Chuna + AT	-	Low<br>Risk of bias (-1)<br>Imprecision (-1)	Low<br>Risk of bias (-1)<br>Imprecision (-1)
Chuna	Chuna +<br>acupressure	Low<br>Risk of bias (-1)<br>Imprecision (-1)	Low<br>Risk of bias (-1)<br>Imprecision (-1)	Low<br>Risk of bias (-1)<br>Imprecision (-1)
Chuna	Cupping	-	Low<br>Risk of bias (-1)<br>Imprecision (-1)	Low<br>Risk of bias (-1)<br>Imprecision (-1)
Chuna	Cupping +<br>acupressure	Low<br>Risk of bias (-1)<br>Imprecision (-1)	-	Low<br>Risk of bias (-1)<br>Imprecision (-1)
Chuna	Fenfluramine	-	Low<br>Risk of bias (-1)<br>Imprecision (-1)	Low<br>Risk of bias (-1)<br>Imprecision (-1)
Chuna	HM	Low<br>Risk of bias (-1)<br>Imprecision (-1)	Low<br>Risk of bias (-1)<br>Imprecision (-1)	Low<br>Risk of bias (-1)<br>Imprecision (-1)
Chuna	HM + AT	-	Moderate<br>Risk of bias (-1)	Moderate<br>Risk of bias (-1)
Chuna	HM + acupressure	-	Low<br>Risk of bias (-1)<br>Imprecision (-1)	Low<br>Risk of bias (-1)<br>Imprecision (-1)
Chuna	None	Low<br>Risk of bias (-1)<br>Imprecision (-1)	Low<br>Risk of bias (-1)<br>Imprecision (-1)	Low<br>Risk of bias (-1)<br>Imprecision (-1)

Chuna + AT	Chuna + acupressure	-	Low<br>Risk of bias (-1)<br>Imprecision (-1)	Low<br>Risk of bias (-1)<br>Imprecision (-1)
Chuna + AT	Cupping	-	Low<br>Risk of bias (-1)<br>Imprecision (-1)	Low<br>Risk of bias (-1)<br>Imprecision (-1)
Chuna + AT	Cupping + acupressure	-	Moderate<br>Risk of bias (-1)	Moderate<br>Risk of bias (-1)
Chuna + AT	Fenfluramine	-	Moderate<br>Risk of bias (-1)	Moderate<br>Risk of bias (-1)
Chuna + AT	HM	-	Low<br>Risk of bias (-1)<br>Imprecision (-1)	Low<br>Risk of bias (-1)<br>Imprecision (-1)
Chuna + AT	HM + AT	-	Moderate<br>Risk of bias (-1)	Moderate<br>Risk of bias (-1)
Chuna + AT	HM + acupressure	-	Low<br>Risk of bias (-1)<br>Imprecision (-1)	Low<br>Risk of bias (-1)<br>Imprecision (-1)
Chuna + AT	None	Low<br>Risk of bias (-1)<br>Imprecision (-1)	-	Low<br>Risk of bias (-1)<br>Imprecision (-1)
Chuna + acupressure	Cupping	-	Low<br>Risk of bias (-1)<br>Imprecision (-1)	Low<br>Risk of bias (-1)<br>Imprecision (-1)
Chuna + acupressure	Cupping + acupressure	-	Low<br>Risk of bias (-1)<br>Imprecision (-1)	Low<br>Risk of bias (-1)<br>Imprecision (-1)
Chuna + acupressure	Fenfluramine	-	Low<br>Risk of bias (-1)<br>Imprecision (-1)	Low<br>Risk of bias (-1)<br>Imprecision (-1)
Chuna + acupressure	HM	-	Low<br>Risk of bias (-1)<br>Imprecision (-1)	Low<br>Risk of bias (-1)<br>Imprecision (-1)
Chuna + acupressure	HM + AT	-	Moderate<br>Risk of bias (-1)	Moderate<br>Risk of bias (-1)
Chuna + acupressure	HM + acupressure	-	Low<br>Risk of bias (-1)<br>Imprecision (-1)	Low<br>Risk of bias (-1)<br>Imprecision (-1)
Chuna + acupressure	None	Low<br>Risk of bias (-1)<br>Imprecision (-1)	Low<br>Risk of bias (-1)<br>Imprecision (-1)	Low<br>Risk of bias (-1)<br>Imprecision (-1)
Cupping	Cupping + acupressure	-	Low<br>Risk of bias (-1)<br>Imprecision (-1)	Low<br>Risk of bias (-1)<br>Imprecision (-1)
Cupping	Fenfluramine	-	Low<br>Risk of bias (-1)<br>Imprecision (-1)	Low<br>Risk of bias (-1)<br>Imprecision (-1)
Cupping	HM	-	Low<br>Risk of bias (-1)<br>Imprecision (-1)	Low<br>Risk of bias (-1)<br>Imprecision (-1)

Cupping	HM + AT	-	Moderate<br>Risk of bias (-1)	Moderate<br>Risk of bias (-1)
Cupping	HM + acupressure	-	Low<br>Risk of bias (-1)<br>Imprecision (-1)	Low<br>Risk of bias (-1)<br>Imprecision (-1)
Cupping	None	Low<br>Risk of bias (-1)<br>Imprecision (-1)	-	Low<br>Risk of bias (-1)<br>Imprecision (-1)
Cupping + acupressure	Fenfluramine	-	Low<br>Risk of bias (-1)<br>Imprecision (-1)	Low<br>Risk of bias (-1)<br>Imprecision (-1)
Cupping + acupressure	HM	-	Low<br>Risk of bias (-1)<br>Imprecision (-1)	Low<br>Risk of bias (-1)<br>Imprecision (-1)
Cupping + acupressure	HM + AT	-	Moderate<br>Risk of bias (-1)	Moderate<br>Risk of bias (-1)
Cupping + acupressure	HM + acupressure	-	Moderate<br>Risk of bias (-1)	Moderate<br>Risk of bias (-1)
Cupping + acupressure	None	-	Low<br>Risk of bias (-1)<br>Imprecision (-1)	Low<br>Risk of bias (-1)<br>Imprecision (-1)
Fenfluramine	HM	Low<br>Risk of bias (-1)<br>Imprecision (-1)	-	Low<br>Risk of bias (-1)<br>Imprecision (-1)
Fenfluramine	HM + AT	-	Moderate<br>Risk of bias (-1)	Moderate<br>Risk of bias (-1)
Fenfluramine	HM + acupressure	-	Moderate<br>Risk of bias (-1)	Moderate<br>Risk of bias (-1)
Fenfluramine	None	-	Low<br>Risk of bias (-1)<br>Imprecision (-1)	Low<br>Risk of bias (-1)<br>Imprecision (-1)
HM	HM + AT	-	Moderate<br>Risk of bias (-1)	Moderate<br>Risk of bias (-1)
HM	HM + acupressure	-	Low<br>Risk of bias (-1)<br>Imprecision (-1)	Low<br>Risk of bias (-1)<br>Imprecision (-1)
HM	None	Moderate<br>Risk of bias (-1)	Low<br>Risk of bias (-1)<br>Imprecision (-1)	Moderate<br>Risk of bias (-1)
HM + AT	HM + acupressure	-	Low<br>Risk of bias (-1)<br>Imprecision (-1)	Low<br>Risk of bias (-1)<br>Imprecision (-1)
HM + AT	None	-	Low<br>Risk of bias (-1)<br>Imprecision (-1)	Low<br>Risk of bias (-1)<br>Imprecision (-1)
HM + acupressure	None	Low<br>Risk of bias (-1)<br>Imprecision (-1)	-	Low<br>Risk of bias (-1)<br>Imprecision (-1)
